# Supplementary material for: Structural Basis of CYRI-B Direct Competition with Scar/WAVE Complex for Rac1
Source: Structure. 2021 Mar 4;29(3):226–237.e4. doi: 10.1016/j.str.2020.11.003 (PMC7955166; doi:10.1016/j.str.2020.11.003)
Supplement: Document S1. Figures S1–S6 and Tables S1 and S2 [file mmc1.pdf]

Structure, Volume *29*

Supplemental Information

Structural Basis of CYRI-B Direct Competition with Scar/WAVE Complex for Rac1

Tamas Yelland, Anh Hoang Le, Savvas Nikolaou, Robert Insall, Laura Machesky, and Shehab Ismail

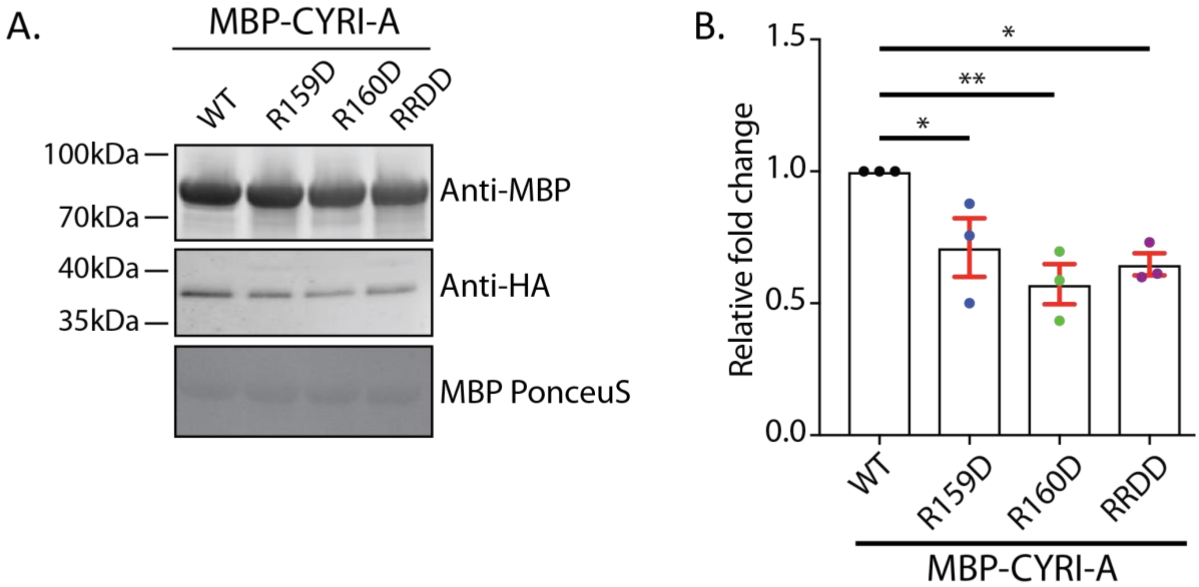

Supplementary Figure 1. Related to Figure 1. CYRI molecules can form heterotypic interactions and the conserved arginine residues play a role in this interaction.

A. Western blot analysis shows immobilised MBP-CYRI-A forms a weak but consistent interaction with HA-CYRI-B. Both single and double mutant of the conserved arginine residues can affect this heterodimerisation.
B. Quantification shows significant decreases in the dimerisation affinity between CYRI-A and CYRI-B when the single or double mutations were introduced.
Data from 3 independent experiments. Mean ± SEM. ANOVA with multiple comparisons. ns = p>0.05, *p<0.05, **p<0.01.

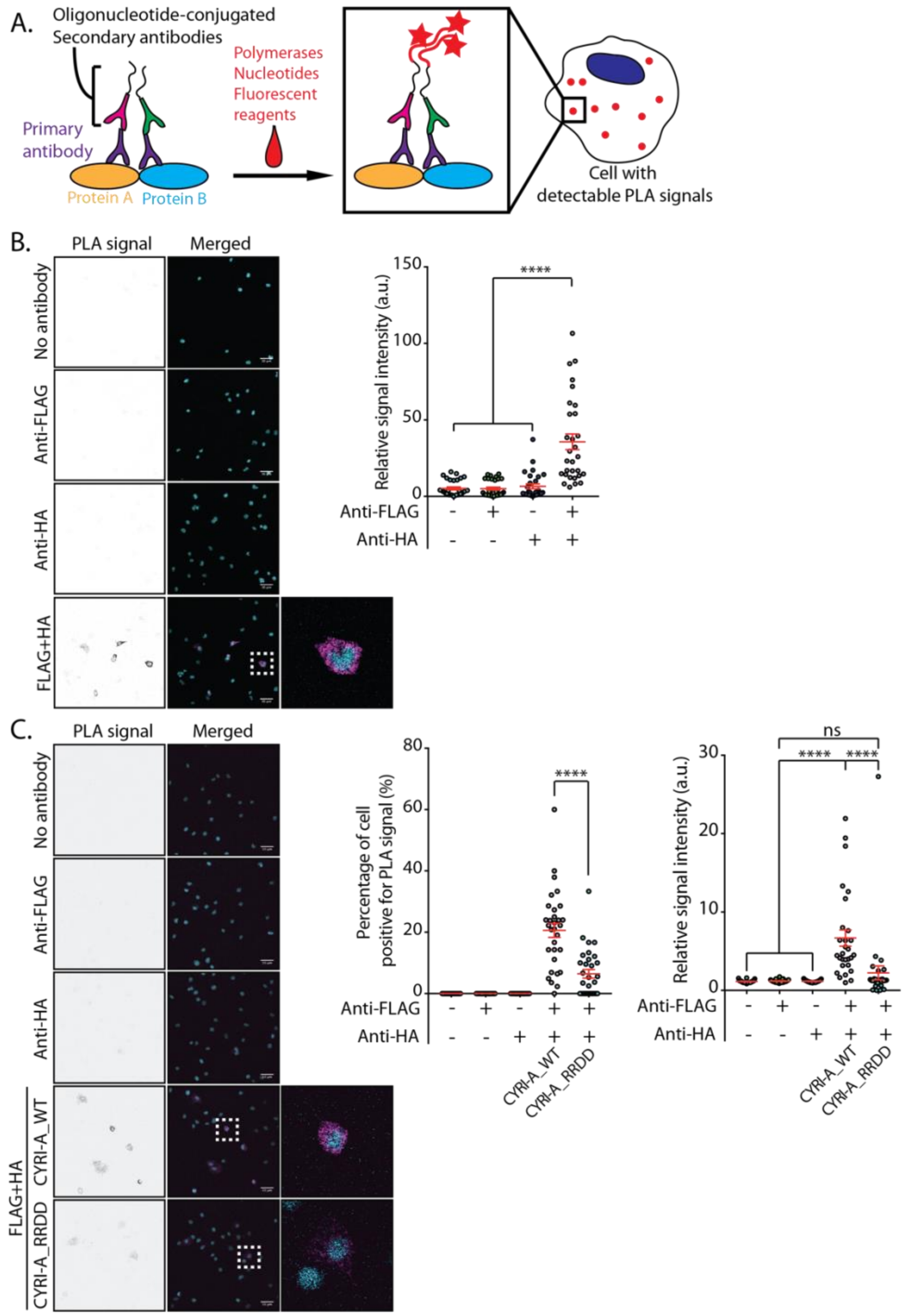

Supplementary Figure 2. Related to Figure 1. CYRI-A and CYRI-B interact with each other in vivo and the interaction is R159/R160 dependent.

A. Schematic representation of how the proximity ligation assay (PLA) works. Any protein within the 40nm proximity can generate the fluorescent PLA signal.
B. COS-7 cells were co-transfected with CYRI-A-FLAG and P17-HA-CYRI-B construct and proceed for a PLA. Only cells expressing both constructs and are probed with both anti-FLAG and anti-HA generated strong PLA signal.
C. Mutating both arginine 159 and 160 to aspartic acid in CYRI-A (RRDD), which are involved in active RAC1 binding, significantly decreases the PLA signal.
Scatter plots of 3 independent experiments. Mean ± SEM. ANOVA with multiple comparisons. ns = $p>0.05$, ****$p<0.0001$.

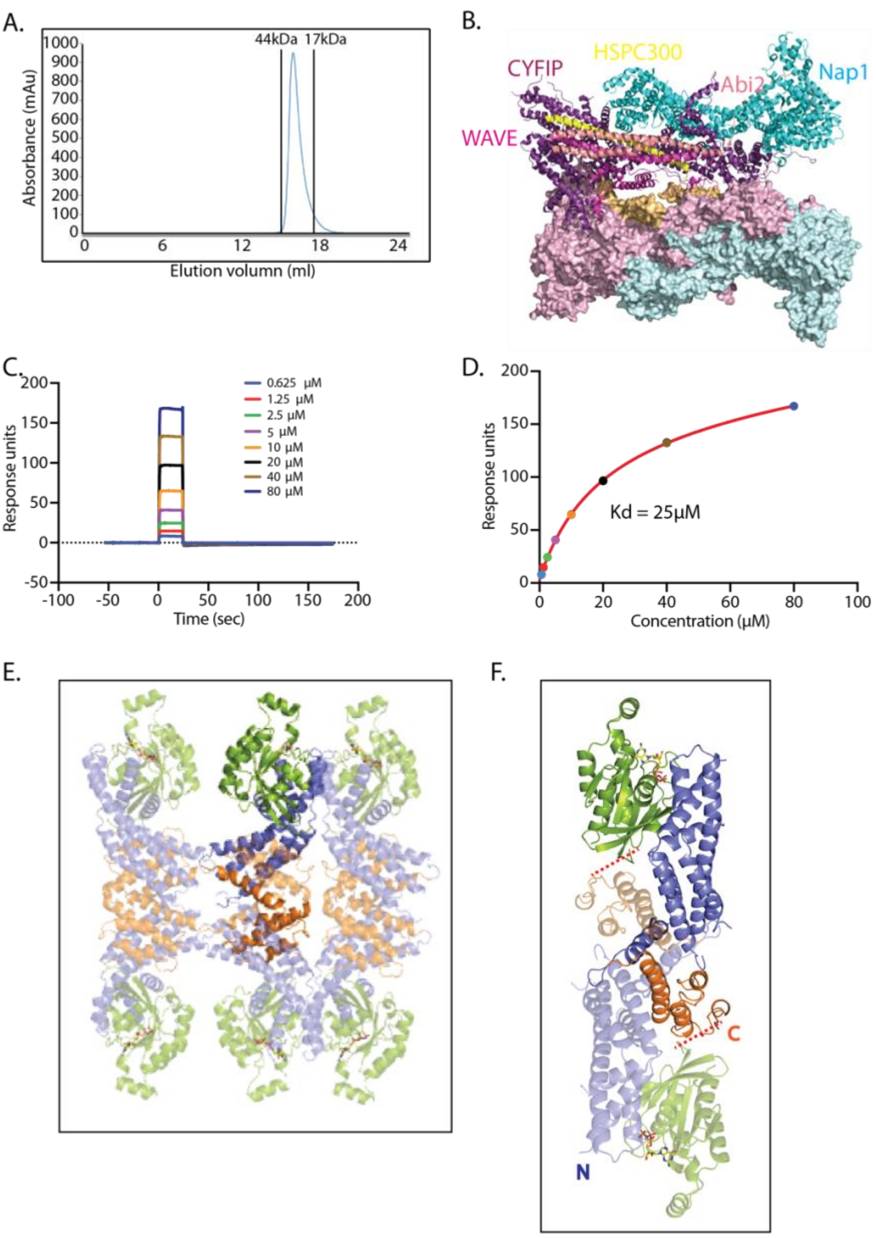

Supplementary Figure 3. Related to Figure 2, 3. CYRI-B oligomerisation, SPR binding curve for Rac1Q61L with MBP-CYRI-B and crystal packing of CYRI-BΔN:Rac1Q61L.

A. CYRI-B could not be detected through analytical size exclusion chromatography, potentially due to its weak affinity. Molecular weight marker elution volumes highlighted.
B. Theoretical Scar/WAVE complex dimers. One Scar/WAVE complex (PDB:3P8C) shown in cartoon form, the second in surface form.
C-D. SPR binding curve and affinity measurement using immobilised MBP-CYRI-B and titrating Rac1Q61L. Titration range from 0.625μM to 80μM Rac1Q61L. Data fitted using T200 analysis software. Figure generated using Prism.
E. Crystal lattice of six CYRI- BΔN:Rac1Q61L molecules. One molecule is shown in solid colour, the remaining five at 50% transparency. Rac1 coloured in green, RBD in blue and the Ratchet subdomain in orange.
F. Linker connecting the C-terminus of CYRI-BΔN to the N-terminus of Rac1Q61L shown in dotted red lines.

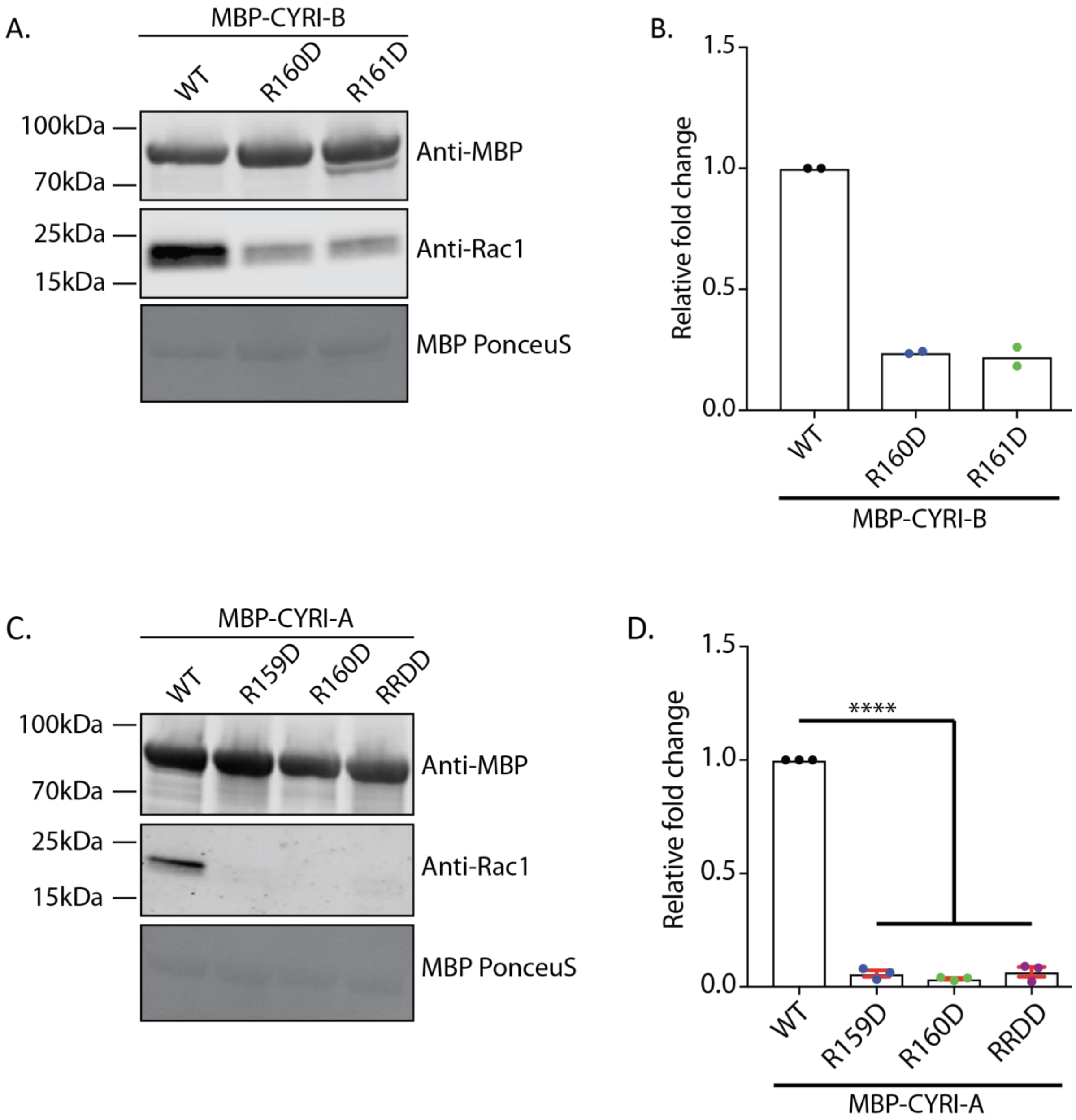

Supplementary Figure 4. Related to Figure 3, 4. The two conserved arginine in CYRI-A and CYRI-B are important for active Rac1 interaction.

A. Pulldown assay using immobilised MBP-CYRI-B shows single arginine mutants could dramatically disrupt their interaction with active Rac1.
B. Quantification shows dramatic decreases in active Rac1 binding to single mutant CYRI-B compared to its WT.
C. Pulldown assay using immobilised MBP-CYRI-A shows both single arginine mutants and double mutant could dramatically disrupt their interaction with active Rac1.
D. Quantification shows dramatic decreases in active Rac1 binding to mutant CYRI-A compared to its WT.

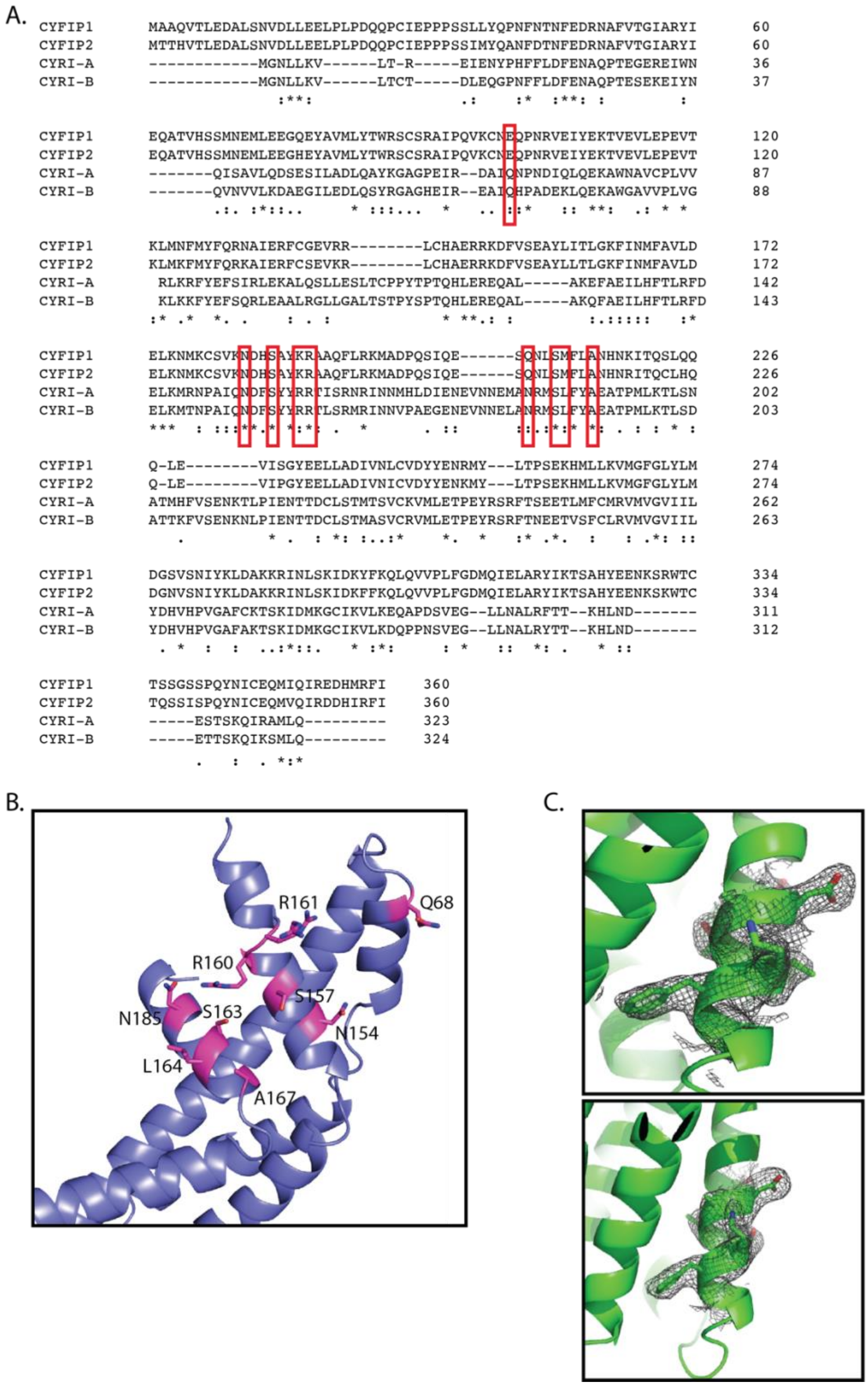

Supplementary Figure 5. Related to Figure 7. Identification of a minimal DUF1394 domain Rac1 binding site.

A. Sequence alignment of human CYFIP1, CYFIP2, CYRI-A and CYRI-B. Residues with conserved biochemical properties at the binding interface are highlighted in red boxes.
B. Conserved residues highlighted on CYRI-B surface in pink and stick form.
C. Representative electron density for both CYRI-BΔN (left) and CYRI-BΔN:Rac1 complex (right). Electron density contoured to 1sigma.

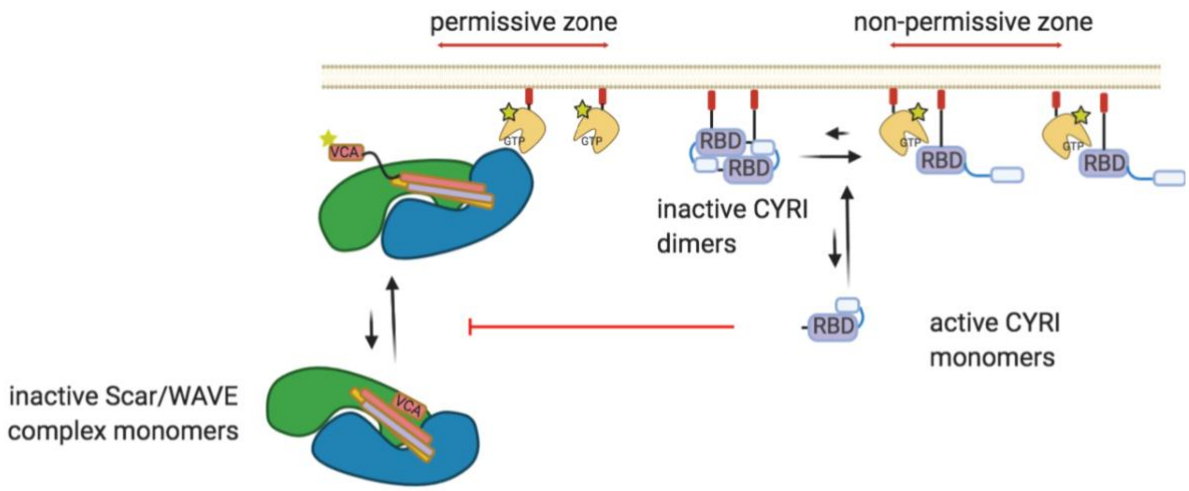

Supplementary Figure 6. Related to Figure 1 to 7. Model for CYRI regulation of active Rac1.

CYRI is recruited by active Rac1 to the plasma membrane. Rac1 binding to the Rac binding subdomain can induce a conformational change displacing the Ratchet subdomain of CYRI. When CYRI binds all active Rac1, a local zone of plasma membrane becomes non-permissive and the Scar/WAVE complex is inhibited. When high local concentrations of CYRI are achieved, an autoinhibited CYRI dimer can be formed, releasing Rac1 to activate the Scar/WAVE complex. Transient interactions between Rac1 and CYRI promote an active feedback loop that can propagate dynamic actin waves, driving protrusions and migration.

	CYRI-BΔN (PDB: 7AJL)	CYRI-BΔN:Rac1 complex (PDB: 7AJK)
Wavelength (Å)	0.97	0.97
Resolution range	41.74 - 2.37 (2.45- 2.37)	55.45 - 3.10 (3.21 - 3.10)
Space group	P 1 21 1	P 62 2 2
Unit cell	44.73 166.68 45.13<br>90.0 112.3 90.0	81.87 81.87 355.87<br>90.0 90.0 120.0
Total reflections	763899	14916
Unique reflections	24723	13492
Multiplicity	23.0 (21.5)	10.6 (11.3)
Completeness (%)	99.94 (100.00)	97.82 (99.77)
Mean I/sigma(I)	25.0 (3.5)	18.7 (3.6)
Wilson B-factor	62.54	88.66
R-merge	7.2 (86.4)	9.2 (66.2)
R-meas	7.5 (90.8)	9.6 (68.1)
CC1/2	0.97 (0.97)	0.99 (0.92)
Reflections used in refinement	24710	13490
Reflections used for R-free	1242	672
R-work	23.9	24.7
R-free	27.2	29.2
No. non-hydrogen atoms	4729	3603
Macromolecules	4568	3558
Solvent	161	12
RMS(bonds)	0.013	0.013
RMS(angles)	1.54	1.54
Ramachandran favoured (%)	95.9	94.7
Ramachandran allowed (%)	4.1	5.1
Ramachandran outliers (%)	0.0	0.2
Rotamer outliers (%)	7.2	6.0
Clashscore	15.6	14.1
Average B-factor	84.5	86.9
Macromolecules	84.9	87.0
Solvent	74.2	65.5

TableS1. Related to Figure 1 and 2. Crystallographic table of statistics

REAGENT or RESOURCE	SOURCE	IDENTIFIER
Oligonucleotides		
CYRI-A-FLAG For:TCAACGGGATCCGCCACCA TGGGAAACTTGCTCAAAGTC	This work/Thermofisher	N/A
CYRI-A-FLAG Rev: TCAACGCTCGAGTTACTTGTCGT CATCGTCTTTGTAGTCCTGAAGCATC GCTCGAATCTG	This work/Thermofisher	N/A
P17-HA-CYRI-B For:TACCCATACGATGTTCCAGAT TACGCTGCTAGCAATTTTTTCCTTG ATTTTGAAAATGCCCAGCC	This work/Thermofisher	N/A
P17-HA-CYRI-B Rev:AGCGTAATCTGGAACATCGTATGGGTACT GAGTGGCCCCTGCTCAAGGTC	This work/Thermofisher	N/A
GST-hRac1Q61L-S41A For: TGACAATTATGCTGCCAATGTTATG	This work/Thermofisher	N/A
GST-hRac1Q61L-S41A Rev: AAGACAGTAGGGATATATTC	This work/Thermofisher	N/A
MBP-mCYRI-B-S157A For: AAATGACTTCGCCTACTACAGAAGAACATTG	This work/Thermofisher	N/A
MBP-mCYRI-B-S157A Rev: TGTATGGCAGGATTTGTC	This work/Thermofisher	N/A
MBP-mCYRI-B-S157R For: AAATGACTTCAGGTACTACAGAAG	This work/Thermofisher	N/A
MBP-mCYRI-B-S157R Rev: TGTATGGCAGGATTTGTC	This work/Thermofisher	N/A
MBP-mCYRI-B-Q153R For: TCCTGCCATACGAAATGACTTCAG	This work/Thermofisher	N/A
MBP-mCYRI-B-Q153R Rev: TTTGTCATCTTGAGCTCATC	This work/Thermofisher	N/A
MBP-HA-mCYRI-B For: CGCGGATCCTATCCGTATGATGT TCCGGATTATGCAATGGGGAATCTTC TTAAAGTTTTGACATGC	This work/IDT	N/A
MBP-HA-mCYRI-B Rev: AAGGAAAAAAGCGGCCGCTTAC TGCAGCATGGACCTAATTTGC	This work/IDT	N/A
Mouse CYRI-BΔN For: CGCGGATCCGCCCAGCCTACA GAGTCTGAGAAG	This work/IDT	N/A
Mouse CYRI-BΔN Rev: CCGGAATTCTTACTATTGCAGCAT GGATTTAATTTGCTTGG	This work/IDT	N/A
MBP-mCYRI-A For: CGTATTGGATCCATGGGAAACTTG CTCAAAGTC	This work/Thermofisher	N/A
MBP-CYRI-A Rev: CGTATTGAAGCTTCTACTGAAGC ATCGCTCGAAT	This work/Thermofisher	N/A
MBP-CYRI-A-R159D For: CAGTTACTATGACAGAACAATAAG TCGTAACCG	This work/Thermofisher	N/A
MBP-CYRI-A-R159D Rev: AAGTCATTCTGAATAGCG	This work/Thermofisher	N/A
MBP-CYRI-A-R160D For: TTACTATCGAGACACAATAAGTCG TAACC	This work/Thermofisher	N/A

MBP-CYRI-A-R160D<br>Rev: CTGAAGTCATTCTGAATAGC	This work/Thermofisher	N/A
MBP-CYRI-A-RRDD<br>For: CAGTTACTATGACGACACAAT AAGTCGTAACCGTATC	This work/Thermofisher	N/A
MBP-CYRI-A-RRDD<br>Rev: AAGTCATTCTGAATAGCG	This work/Thermofisher	N/A

TableS2. Related to Key Resource Table. Oligonucleotides
