# Supplementary material for: Structural Basis of CYRI-B Direct Competition with Scar/WAVE Complex for Rac1
Source: Structure. 2021 Mar 4;29(3):226–237.e4. doi: 10.1016/j.str.2020.11.003 (PMC7955166; doi:10.1016/j.str.2020.11.003)
Supplement: Document S2. Article plus Supplemental Information [file mmc3.pdf]

Structure

Article

Structural Basis of CYRI-B Direct Competition with Scar/WAVE Complex for Rac1

Graphical Abstract

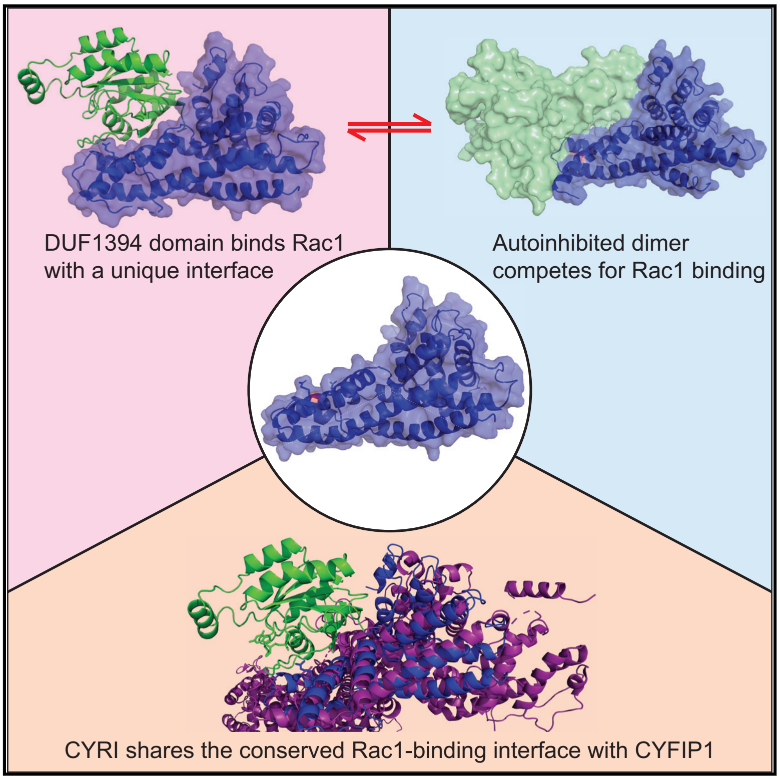

Highlights

- CYRI-B structure reveals homology to CYFIP of the Scar/WAVE complex
- CYRI-B shares a conserved interface with CYFIP for Rac1 interaction
- CYRI proteins form autoinhibited dimers that compete for Rac1 binding
- Rac1-CYFIP model shows potential steric clashes of RAC1 with CYFIP

Authors

Tamas Yelland, Anh Hoang Le, Savvas Nikolaou, Robert Insall, Laura Machesky, Shehab Ismail

Correspondence

laura.machesky@glasgow.ac.uk (L.M.), shehab.ismail@glasgow.ac.uk (S.I.)

In Brief

Yelland et al. determined the structure of CYRI-B alone and in complex with Rac1Q61L. These structures show that the binding interface is conserved with CYFIP of the Scar/WAVE complex providing an explanation for CYRI competition. Finally, CYRI proteins form autoinhibited dimers generating additional complexity to CYRI function.

Yelland et al., 2021, Structure *29*, 226–237
March 4, 2021 © 2020 The Authors. Published by Elsevier Ltd.
https://doi.org/10.1016/j.str.2020.11.003

CellPress
OPEN ACCESS

Structure

Article

Structural Basis of CYRI-B Direct Competition with Scar/WAVE Complex for Rac1

Tamas Yelland,[1,4] Anh Hoang Le,[1,4] Savvas Nikolaou,[1] Robert Insall,[1,2] Laura Machesky,[1,2,*] and Shehab Ismail[1,2,3,5,*]
[1]CRUK- Beatson Institute, Glasgow G61 1BD, UK
[2]Institute of Cancer Sciences, University of Glasgow, Glasgow G61 1BD, UK
[3]Department of Chemistry, KU Leuven, Celestijnenlaan 200G, 3001 Heverlee, Belgium
[4]These authors contributed equally
[5]Lead Contact
*Correspondence: laura.machesky@glasgow.ac.uk (L.M.), shehab.ismail@glasgow.ac.uk (S.I.)
https://doi.org/10.1016/j.str.2020.11.003

SUMMARY

Rac1 is a major regulator of actin dynamics, with GTP-bound Rac1 promoting actin assembly via the Scar/WAVE complex. CYRI competes with Scar/WAVE for interaction with Rac1 in a feedback loop regulating actin dynamics. Here, we reveal the nature of the CYRI-Rac1 interaction, through crystal structures of CYRI-B lacking the N-terminal helix (CYRI-BΔN) and the CYRI-BΔN:Rac1Q61L complex, providing the molecular basis for CYRI-B regulation of the Scar/WAVE complex. We reveal CYRI-B as having two subdomains - an N-terminal Rac1 binding subdomain with a unique Rac1-effector interface and a C-terminal Ratchet subdomain that undergoes conformational changes induced by Rac1 binding. Finally, we show that the CYRI protein family, CYRI-A and CYRI-B can produce an autoinhibited hetero- or homodimers, adding an additional layer of regulation to Rac1 signaling.

INTRODUCTION

Regulation of the cytoskeleton has a direct impact on cellular shape, polarity, migration, and homeostasis. Actin is the key effector protein shaping cytoskeleton dynamics at membrane interfaces (Krause and Gautreau 2014). Recently, a novel effector of Rac1, Fam49B also known as CYRI-B (CYFIP-related Rac interactor), has been reported to bind to active Rac1 and oppose the activation of the Scar/WAVE complex (Fort et al., 2018). It was described as a local inhibitor of branched actin assembly, and is thought to be recruited by the activating signal-active Rac1-and suppresses Scar/WAVE activity (Fort et al., 2018). This local inhibition buffers the actin polymerization process at the leading edge (Fort et al., 2018), increases membrane dynamics, suppresses T cell activation (Shang et al., 2018) and can promote resistance to *Salmonella* infection (Yuki et al., 2019).

CYRI-B, its isoform CYRI-A and the Scar/WAVE complex all possess an evolutionarily conserved Rac1 binding domain (RBD) termed the DUF1394 domain. Based on this homology, CYRI's suppression of Rac1-mediated actin polymerization was proposed to be through direct competition with the Scar/WAVE complex (Fort et al., 2018). Nevertheless, the structural basis of this interaction and the interplay with Scar/WAVE binding are not known.

The Scar/WAVE complex is composed of five subunits; CYFIP, NCKAP1, Scar/WAVE, ABI, and HSPC300. It has been proposed that the Scar/WAVE complex undergoes an activating conformational change (Chen et al., 2010) induced by its recruitment to the plasma membrane through interactions with negatively charged phospholipids and binding to "active" GTP-bound Rac1 (Chen et al., 2010; Hoeller et al., 2016; Veltman et al., 2012). Activation of the Scar/WAVE complex releases the C-terminal VCA region of Scar/WAVE, promoting binding to and activation of Arp2/3, leading to branched actin polymerization (Davidson and Insall, 2011). While Rac1 binding to Scar/WAVE and CYRI has been biochemically detected, little is known about the binding interface. Based on the crystal structure of Scar/WAVE, a Rac1 binding site on CYFIP termed the "A" site was proposed and validated using pulldown experiments (Chen et al., 2010). A recent cryo-EM structure of the Scar/WAVE complex fused to Rac1 also revealed a secondary Rac1 interaction site, which has been named the "D" site (Chen et al., 2017). Nevertheless, mutagenesis studies revealed that the "A" site was the most potent regulator of lamellipodia activity in cells (Chen et al., 2017; Schaks et al., 2018). Importantly, the atomic level interaction and structural basis of Rac1-mediated activation and release of the VCA region remains unresolved.

Here, we present the crystal structures of mouse CYRI-B (residues 26–324 hereafter termed CYRI-BΔN) and human CYRI-BΔN in complex with the constitutively active mutant Rac1Q61L.GppNHp. This is the first structure of a DUF1394 domain in complex with Rac1 and provides the structural details dictating Rac1 specificity for this module. Based on these structures, we define a novel Rac1 interacting domain and provide clues to how CYRI can directly and locally compete with the Scar/WAVE

 © 2020 The Authors. Published by Elsevier Ltd.
This is an open access article under the CC BY license (http://creativecommons.org/licenses/by/4.0/).

Table 1. Crystallographic Table of Statistics

	CYRI-BΔN (PDB: 7AJL)	CYRI-BΔN: Rac1 Complex (PDB: 7AJK)
Wavelength (Å)	0.97	0.97
Resolution range	41.74–2.37 (2.45–2.37)	55.45–3.10 (3.21–3.10)
Space group	P 1 21 1	P 62 2 2
Unit cell	44.73 166.68 45.13 90.0 112.3 90.0	81.87 81.87 355.87 90.0 90.0 120.0
Total reflections	763899	14,916
Unique reflections	24,723	13,492
Multiplicity	23.0 (21.5)	10.6 (11.3)
Completeness (%)	99.94 (100.00)	97.82 (99.77)
Mean I/sigma(I)	25.0 (3.5)	18.7 (3.6)
Wilson B-factor	62.54	88.66
R-merge	7.2 (86.4)	9.2 (66.2)
R-meas	7.5 (90.8)	9.6 (68.1)
CC1/2	0.97 (0.97)	0.99 (0.92)
Reflections used in refinement	24,710	13,490
Reflections used for R-free	1,242	672
R-work	23.9	24.7
R-free	27.2	29.2
No. non-hydrogen atoms	4,729	3,603
Macromolecules	4,568	3,558
Solvent	161	12
RMS(bonds)	0.013	0.013
RMS(angles)	1.54	1.54
Ramachandran favored (%)	95.9	94.7
Ramachandran allowed (%)	4.1	5.1
Ramachandran outliers (%)	0.0	0.2
Rotamer outliers (%)	7.2	6.0
Clashscore	15.6	14.1
Average B-factor	84.5	86.9
Macromolecules	84.9	87.0
Solvent	74.2	65.5

complex. Furthermore, the complex structure leads to a model for how Rac1 activates the Scar/WAVE complex. Finally, we show that CYRI proteins can homo- or heterodimerize. Dimerization uses the same interface as Rac1 binding, leading us to propose an autoinhibition mechanism for the regulation of CYRI.

RESULTS

Crystal Structure of CYRI-BΔN

CYRI proteins are evolutionarily conserved and show low sequence identity (~18% in humans) to the Scar/WAVE complex subunit CYFIP, which shares a DUF1394 domain. As there is no structural data for CYRI, we set out to solve its crystal structure. Despite mouse CYRI-A and CYRI-B sharing 80% sequence identity, significant differences in solubility were observed, with the latter showing better solubility. We, therefore, focused our crystallographic efforts on CYRI-B. Initial crystal screens were set up for the full-length CYRI-B and although crystals were obtained, they diffracted poorly and resisted optimization. To obtain crystals capable of diffracting to high resolution, the N-terminal residues (1–25) involved in membrane binding were truncated, but the entire DUF1394 domain (residues 26–324) was retained. We solved the crystal structure to 2.37Å resolution (Table 1), using experimental phasing, as molecular replacement using CYFIP was not successful.

The structure of each CYRI-BΔN monomer consists of 12 alpha helices packed into two bundles running perpendicular to each other to produce an L-shape fold (Figure 1A). The asymmetric unit contains two molecules of CYRI-BΔN with each monomer "slotting" against the other to form a compact dimer (Figure 1B). The dimer interface produces a large contact area of 1524Å^2, yet there are relatively few bonding contacts (Figures 1C, 1D) between the CYRI monomers. Briefly, R161 form a hydrogen bond with A192, R165 and M166 binds to Q324, I168 with R320, N169 with S321 and Q324. With such a low density of bonding interactions between each monomer, suggests that despite a large binding interface the potential dimerization may be weak.

Using the DALI server (http://ekhidna2.biocenter.helsinki.fi/dali/), we compared our structure to all PDB entries. This showed that the only protein having reasonable structural homology (RMSD <5Å for the entire protein) with CYRI-B is CYFIP1 (Figure 1E). Remarkably, given the divergence in sequence, the two proteins overlay with an RMSD of 2.7Å for all Cα atoms, and 1.9Å for residues 26–214 of CYRI-B. The region of greatest divergence corresponds to the C-terminal helical bundle where the alignment of residues 215–324 of CYRI-B to CYFIP gives an RMSD of 5.1Å for Cα atoms due to an insertion of an antiparallel β-hairpin in CYFIP (Figure 1E). Based on the structural alignment, we define CYRI-B DUF1394 domain as a module comprising two helical bundles in an L-shape fold (Figure 1F).

CYRI-B runs as a monomer on size exclusion chromatography (Figure S3A). Thus to test if the CYRI-B dimer observed in the asymmetric unit can occur in solution and if it can heterodimerize with CYRI-A, we used pulldown assays, which revealed a weak but reproducible interaction between MBP-CYRI-A or CYRI-B with HA-CYRI-B (Figures S1A and Figure 4B). Since the structure of the CYRI-B:CYRI-A heterodimer is not available to validate the heterodimer interface, we mutated residues on CYRI-A based on the CYRI-B homodimer structure. Both single and double mutation of the two conserved arginine residues in CYRI-A reduce the dimerization by up to 50% (Figures S1A and S1B). To investigate if the heterodimerization could occur in a cellular context, we performed proximity ligation assay (PLA). A positive signal was strongly observed with wild-type CYRI-A but is significantly reduced in the RRDD mutant, confirming the possibility of heterodimerization in cells (Figures S2B and S2C).To determine whether the CYRI dimer interface could be conserved with the Scar/WAVE complex, we overlaid the crystal structure of the Scar/WAVE complex (PDB: 3P8C) with each monomer of the CYRI-B dimer (Figure S3B). The resulting structure shows minimal steric clashes between each Scar/WAVE complex

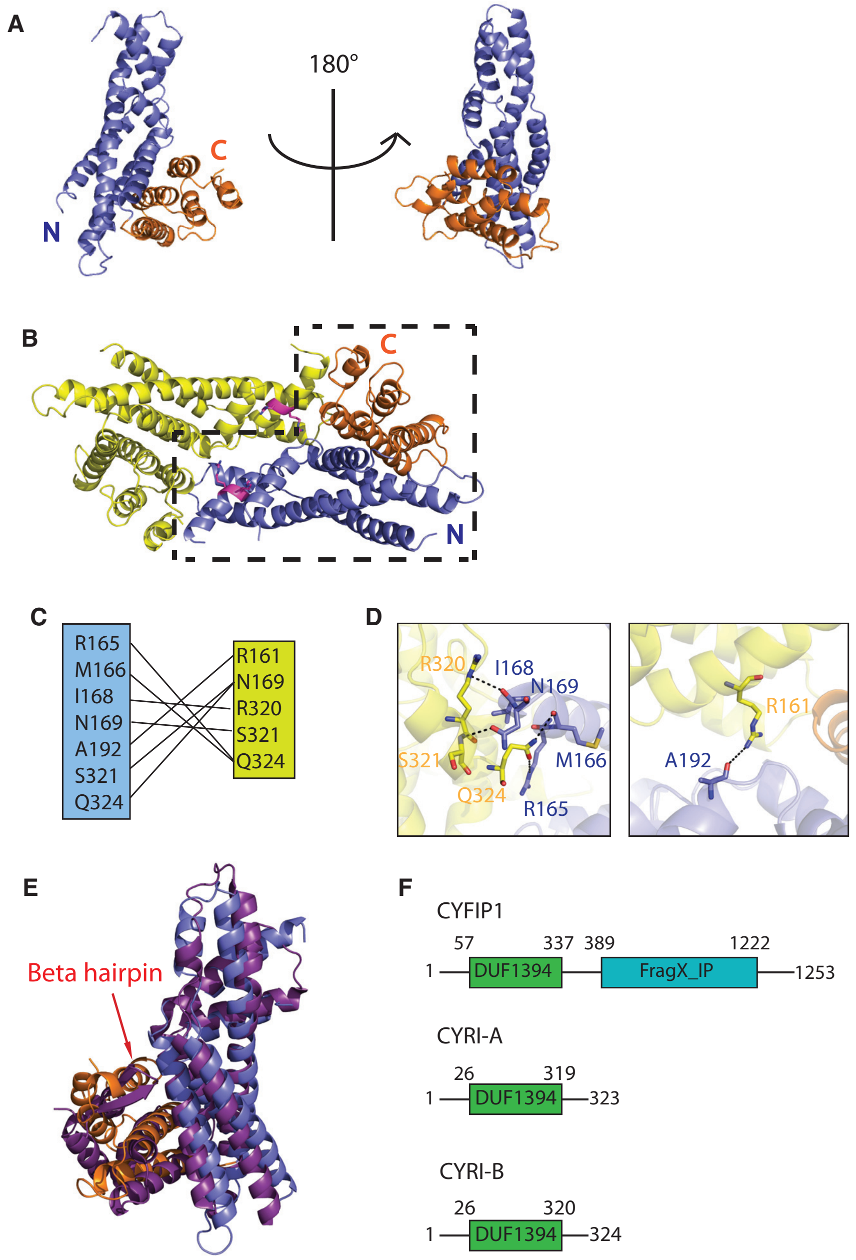

Figure 1. Crystal Structure of CYRI-BΔN

(A) Crystal structure of CYRI-BΔN monomer with a 180° rotation. The two helical bundles are highlighted in different colors: the N-terminal residues (26–204) in blue and the C-terminal residues (205–324) in orange. On the left, the L-shape is evident with the smaller C-terminal helical bundle packed against the larger N-terminal helical bundle.

(B) Crystal structure of CYRI-BΔN contains a dimer within the asymmetric unit. One CYRI-BΔN monomer colored in yellow, the second boxed in black dashed lines and colored with the N-terminal residues (26–204) in blue and the C-terminal residues (205–324) in orange. Arginine residues R160 and R161 mutated in dimerization experiments highlighted in pink.

(C) Table highlighting hydrogen bonding interactions at the CYRI-B dimer interface.

(D) Hydrogen bond interactions involved in CYRI dimer interface are illustrated. Bonding interactions are shown in black dashed lines.

(E) Overlay of CYRI-BΔN colored with N-terminal (residue 26–204) in blue and C-terminal (residue 205–324) in orange with the DUF1394 domain of CYFIP1 (in purple) containing the β-hairpin insert highlighted with red arrow.

(F) Domain structure of CYFIP1, CYRI-A, and CYRI-B.

suggesting that this dimerization may be a conserved feature of DUF1394 containing proteins. In line with this observation, it has been reported that the Scar/WAVE complex can form higher-order oligomers (Pipathsouk et al., 2019).

Rac1 Binding to CYRI-B Is Mediated through a Unique Interface with the Switch-I Loop

To understand how Rac1 interacts with DUF1394 domains in general and CYRI-B specifically, we determined the structure of the complex between CYRI-BΔN and Rac1Q61L. Attempts to co-crystallize CYRI-BΔN and Rac1Q61L.GppNHp were not successful, most likely due to the low binding affinity (measured dissociation constant (Kd) of 25μM – Figures S3C and S3D). To overcome this challenge, a 10-residue linker (GSAGSAGSAG) at CYRI-BΔN's C-terminus was identified that successfully produced crystals of the complex that diffracted to 3.1Å resolution (Figure 2A, Table 1). The crystal structure contains one copy of the CYRI-BΔN in complex with one Rac1Q61L molecule in the asymmetric unit where Rac1Q61L binds in trans with CYRI-BΔN from a symmetry mate (Figures S3E and S3F). Examination of the CRYI-BΔN in complex with Rac1Q61L shows that binding is primarily mediated through extensive contacts between the N-terminal helical bundle of CYRI-BΔN and the switch-I loop (residues 25–39) of Rac1Q61L along with residues on the adjacent β-strand but no contribution from the switch-II loop of Rac1 (Figure 2B), resulting in a large interface of 1097Å^2.

As the DUF1394 domain shows no sequence homology to other Rac1 effectors, we compared the CYRI-BΔN:Rac1Q61L complex with other Rac1 effector complexes; Rac1: Arfaptin (PDB: 1I4T), Rac1: pPhox67 (PDB: 1E96) and Rac1: PRK (PDB: 2RMK) (Figures 2C, 2D, and 2E). Although binding of pPhox67 to Rac1 utilizes the switch-I of Rac1, additional contacts are also made through Q162, A159 L160, and Q162 of Rac1 (Lapouge et al., 2000), which are not observed in CYRI-B binding. The interface with PRK and Arfaptin is mediated through fewer switch-I contacts than CYRI-BΔN but both make additional interactions through the switch-II loop of Rac1 (Modha et al., 2008; Tarricone et al., 2001) that are also not observed in CYRI-BΔN. This results in a different switch-I conformation in Rac1 when bound to CYRI-BΔN compared with Arfaptin and

CellPress
OPEN ACCESS

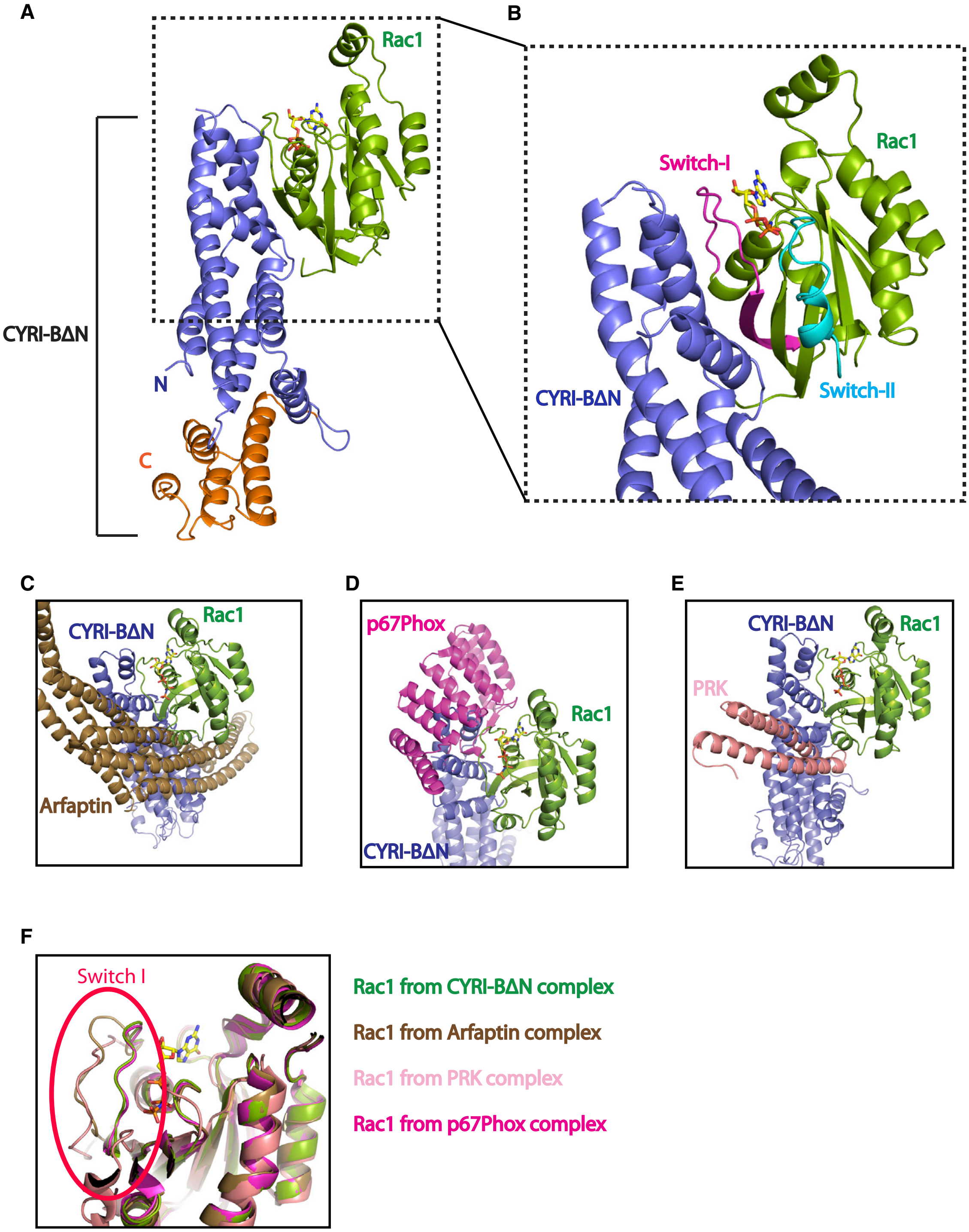

Figure 2. Crystal Structure of the CYRI-BΔN: Rac1Q61L Complex

(A) Structure of the CYRI-BΔN: Rac1Q61L complex. Rac1Q61L is shown in green with bound GppNHp in stick form in yellow. The N-terminal residues (26–204) of CYRI-BΔN are shown in blue and the C-terminal residues (205–324) in orange.

(B) The interface between CYRI-BΔN (in blue) and Rac1 (in green) is mediated by the switch-I loop (in pink) of Rac1Q61L. The binding interface with CYRI has no contribution from the Rac1's switch-II loop (highlighted in cyan).

(C) Comparison of CYRI-BΔN (blue) binding to Arfaptin (brown, PDB: 1I4T).

(D) Comparison of CYRI-BΔN (blue) binding to p67Phox (pink, PDB: 1E96).

(E) Comparison of CYRI-BΔN (blue) binding to PRK (salmon, PDB: 1E96).

(F) Comparison of the switch-I loop of Rac1 when bound to different effectors. Green for Rac1 bound to CYRI-BΔN, brown for Rac1 bound to Arfaptin, pink for Rac1 bound to p67Phox and salmon for Rac1 bound to PRK.

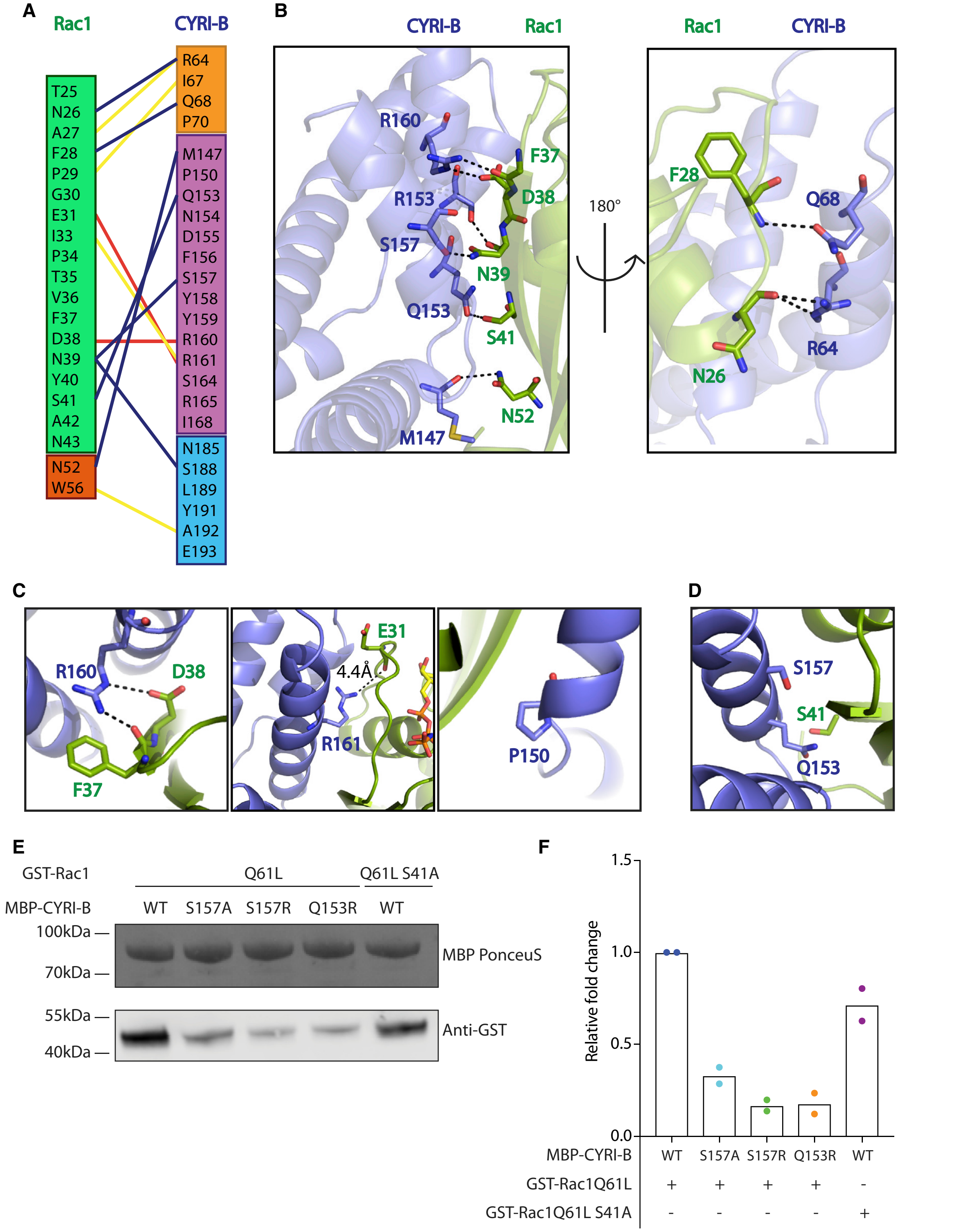

Figure 3. Validation of the CYRI-BΔN: Rac1Q61L Complex

(A) Interaction map between Rac1Q61L and CYRI-B. Black lines are hydrogen bonds, red lines are salt bridges and yellow lines are hydrophobic contacts.

(B) Hydrogen bonding (dashed lines) between CYRI-B (blue) and Rac1 (green).

(legend continued on next page)

CellPress
OPEN ACCESS

PRK (Figure 2F). Thus, CYRI-B binds to Rac1 with a unique interface.

While the interface between CYRI-B and Rac1 is unique, its contact with the switch-I loop of Rac1 explains the specificity for active GTP-bound Rac1. Binding is mediated through a combination of polar and hydrophobic interactions. Despite the size of the interface, there are relatively few interactions (Figures 3A and 3B). The R160 residue of CYRI-B forms a salt bridge with the side chain of Rac1 D38 and an additional hydrogen bond with the main-chain carbonyl group of Rac1 F37. Q153 and R64 of CYRI-B form a hydrogen bond with S41 and N26 of Rac1, respectively. The main-chain carbonyl of M147 forms a hydrogen bond with N52 of Rac1 and S157 of CYRI-B peptide carbonyl forms a hydrogen bond with the side chain of N39 of Rac1. This relatively large interface, has low interaction density explaining the modest affinity between Rac1 and CYRI-B. Rac1 P29S is a clinically important mutation (Hodis et al., 2012; Krauthammer et al., 2012), as it allows Rac1 to undergo spontaneous nucleotide exchange in the absence of a GEF, producing a shift to active Rac1 (Davis et al., 2012). It has been reported that the dual mutation P29S Q61L increases the affinity of Rac1 to CYRI-B and not other effectors such as the CRIB domain (Fort et al., 2018; Whitelaw et al., 2019). As P29 is located at the binding interface between Rac1 and CYRI-B (Figure 3A), one could speculate that mutation to a serine could result in additional hydrogen bonds, perhaps with R161 to increase the affinity of the complex.

Previously we have shown that CYRI-B R160 and R161 are important in binding to Rac1 (Fort et al., 2018) and another report indicates that P150 is also involved (Yuki et al., 2019). Examination of the complex shows that R160 forms hydrogen bonds with D38 and mutating R160 to an aspartic acid would introduce a repulsive charge-charge interaction and thus disrupt complex formation (Figure S4A). The R161 residue is adjacent to Rac1's switch-I loop and although the distance to the nearest Rac1 residue is 4.4Å, a reversal of the charge will also disrupt the interface (Figures S4A and S4B). We also showed this to be the case for CYRI-A (Figures S4C and S4D). P150 is in close proximity to bound Rac1 and although not directly involved in making contacts, a mutation to arginine would introduce steric clashes, inhibiting complex formation (Yuki et al., 2019) (Figure 3C). To further validate the complex, we rationally introduced mutations at the interface of the complex, S157 on CYRI-B was mutated to both alanine and arginine, Q153 was mutated to arginine and S41 on Rac1 was mutated to alanine (Figure 3D). Following pulldown experiments, all mutations resulted in reduced binding (Figures 3E and 3F). We conclude that the DUF1394 domain of CYRI-B binds Rac1 through a unique effector-binding interface and we identify residues involved in this interaction, suggesting a model for how the DUF1394 domain specifically interacts with active Rac1.

Rac1 Sequestration by CYRI Is Regulated through Autoinhibited CYRI Dimers

Overlaying CYRI-BΔN:Rac1Q61L complex structure with that of the CYRI dimer, we can see that the two directly compete for the same binding interface (Figure 4A). This raises the possibility that CYRI dimers autoinhibit Rac1 binding and regulate CYRI functionality. To test if the dimer interface competes with Rac1 binding, the pulldown assays were repeated in the presence of increasing levels of active Rac1. As seen in Figures 4B, 4C, and 4D, both homo and heterodimers of CYRI are disrupted by the addition of active Rac1.

Interestingly, following the overlay of the CYRI-BΔN structure with that of the CYRI-BΔN:Rac1Q61L complex, we can see that the C-terminus of CYRI-BΔN undergoes a conformational change such that it undergoes a domain swap with a symmetry mate (Figure 5A, Video S1). The conformational change is induced through a steric clash between residue Q2 of Rac1 and Y305 of CYRI-B (Figure 5A). The domain-swapped C-terminal bundles occupy the same site as in the CYRI-BΔN structure but is shifted by 5Å, allowing Rac1 to bind without steric clashes and might act as an alternative mechanism for CYRI dimerization (Figure 5C). When overlaying CYRI-BΔN structure with that of the Scar/WAVE complex (PDB: 3P8C) (Figure 5D), no steric clash with Rac1 is observed. This implies that the steric clash with Rac1 is either specific to CYRI-B or that the crystal structure of the Scar/WAVE complex has captured a conformation where no Rac1 clashes would occur. However, as Rac1 failed to induce dimerization of CYRI-B in solution, we conclude that the dimerization mechanism via domain swapping is most likely a crystallization artifact. Nonetheless, the C-terminus of CYRI does undergo a conformational change induced through Rac1 binding. Therefore, assuming that CYRI evolved as a fragment of CYFIP, which retained Rac1 binding activity, it has acquired new and defining features, distinguishing it from a pure Rac1 binding module. The destabilization of intramolecular contacts through GTPase binding is a common feature in Rho family effector complexes and is seen in other proteins, such as the formins (Kühn et al., 2015), and IRSp53 (Kast et al., 2014). As the C-terminal alpha-helical bundle (residues 215–324) is not involved in binding to Rac1, and Rac1 binding interactions are mediated through the N-terminal alpha-helical bundle (residues 26–215), we conclude that the CYRI-BΔN DUF1394 domain contains two subdomains. We define these subdomains as the N-terminal Rac1 binding subdomain (RBD) and the C-terminal Ratchet subdomain (Figure 5A), as it undergoes a conformational change upon Rac1 binding. Binding of active Rac1 to CYRI-BΔN destabilizes the contacts between the Ratchet subdomain and RBD through steric clashes. The precise biological role of the Rac1-induced conformational change to the Ratchet subdomain in CYRI remains elusive.

(C) Previously identified residues important for CYRI-B:Rac1 interaction are highlighted. R160 of CYRI-B (blue) forms tight contacts with D38 and F37. R161 of CYRI-B is adjacent to Rac1's switch-I loop. Distance to closest Rac1 residue (E31 – 4.4Å) is highlighted by a black dashed line. P150 of CYRI-B are in close proximity of Rac1 (green).

(D) Residues selected for mutagenesis to validate the CYRI-BΔN: Rac1Q61L complex.

(E) MBP-tagged CYRI-B wild-type (WT) or mutants (S157A, S157R, Q153R) were immobilized onto MBP beads. Purified GST-tagged Rac1 Q61L or Q61L S41A mutant were in solution. Both mutations on CYRI-B and on Rac1 dramatically affect the binding between the proteins.

(F) Quantification from two independent experiments shows dramatic decreases in band intensity of the mutants.

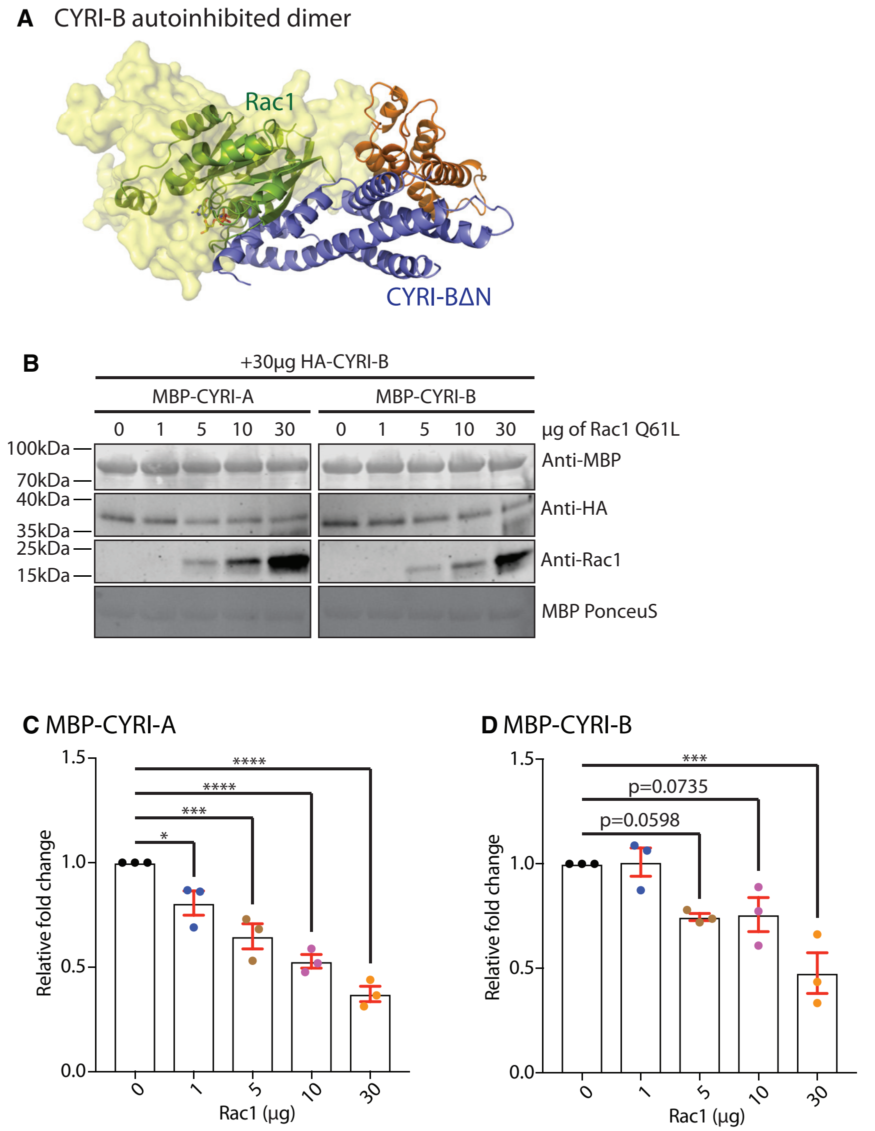

Figure 4. CYRI Dimers Autoinhibit Rac1 Binding

(A) Overlay of CYRI-B dimers with one molecule shown in cartoon form with the RBD in blue and the Ratchet subdomain in orange. The second molecule is shown in yellow surface representation at 50% transparency. The bound Rac1 molecule is shown in green cartoon form. CYRI dimers directly compete with Rac1 binding, thus autoinhibit CYRI.

(B) Pulldown showing the increasing level of Rac1 competes with CYRI homo- and heterodimerization. Immobilized MBP-tagged CYRI-A or CYRI-B forms dimers with HA-tagged CYRI-B, which is reduced with increasing levels of Rac1.

(C and D) Quantification of Rac1 competition with CYRI dimers in (B). Mean ± SEM. ANOVA with multiple comparisons. **$p < 0.01$, ***$p < 0.001$, ****$p < 0.0001$.

Molecular Basis for Rac1 Specificity of DUF1394 over Other GTPases

It remains a major question in the field how CYRI and by analogy CYFIP proteins interact specifically with Rac1, while WASP proteins can bind either Rac1 or Cdc42. We, therefore, performed a sequence alignment for Rac1 against Cdc42 and other less related GTPases RhoA and RND1 (Figure 6A). Together with a structural comparison of Cdc42 (PDB: 4JS0) with our complex, the basis for selectivity becomes apparent. Residues A27, G30 in Rac1 are lysine and serine in Cdc42; both residues result in steric clashes with CYRI-B (Figures 6B–6E). S41 in Rac1 is an alanine in Cdc42 resulted in breaking a hydrogen bond with Q153 of CYRI-B whereas W56 is a phenylalanine residue in Cdc42, which reduces the extent of hydrophobic interactions. Mutation of Rac1 S41A used to validate the interface also serves to validate Rac1 selectivity, as this mutant mimics Cdc42. This mutation disrupts binding by ~30% (Figure 3D, E). Sequence comparison to more distantly related members of the Rho GTPase family RhoA and RND1 (Figure 6A) demonstrates even greater levels of sequence variation and together provides the basis for Rac1 selectivity to CYRI. As CYRI has structural similarity to CYFIP, it is reasonable to extrapolate that Rac1 would bind to CYFIP with a similar binding mode. Therefore, it is tempting to reason that the sequence variations explaining Rac1 selectivity to CYRI may also explain the selective activation of the Scar/WAVE complex by Rac1. For example, a Rac1 mutation A27K (a Cdc42 sequence variation) could induce steric clashes with CYRI-B (Figure 6B), mimicking cdc42, and this could prevent it from binding to the "A" site of CYFIP and activating the Scar/WAVE complex.

In summary, the DUF1394 domain is a Rac1 selective binding module, and sequence alignments with other Rho family GTPases provide us with evidence to explain this binding specificity.

CYRI and CYFIP Have a Conserved, but Distinct Rac1 Interface

Both CYRI-A and CYRI-B can bind to active Rac1 (Figure S4). Examination of the residues involved in this interaction (Figure 6A) shows that all of them are conserved between CYRI isoforms with the exception of M147N; however, this amino acid's side chain is not involved in Rac1 contacts, as binding is mediated by a main-chain hydrogen bond (Figure 3B). Furthermore, mutation of residues at the interface between CYRI and Rac1 (R159D, R160D in CYRI-A and R160D, R161D in CYRI-B) showed comparable reductions in Rac1 binding between the two CYRI isoforms (Figure S4). Thus, Rac1 binding is conserved between CYRI isoforms and is likely to involve the same interface.

As there is limited structural data for Rac1 binding to the "A" site of the Scar/WAVE complex, we sought to use our complex as a

CellPress
OPEN ACCESS

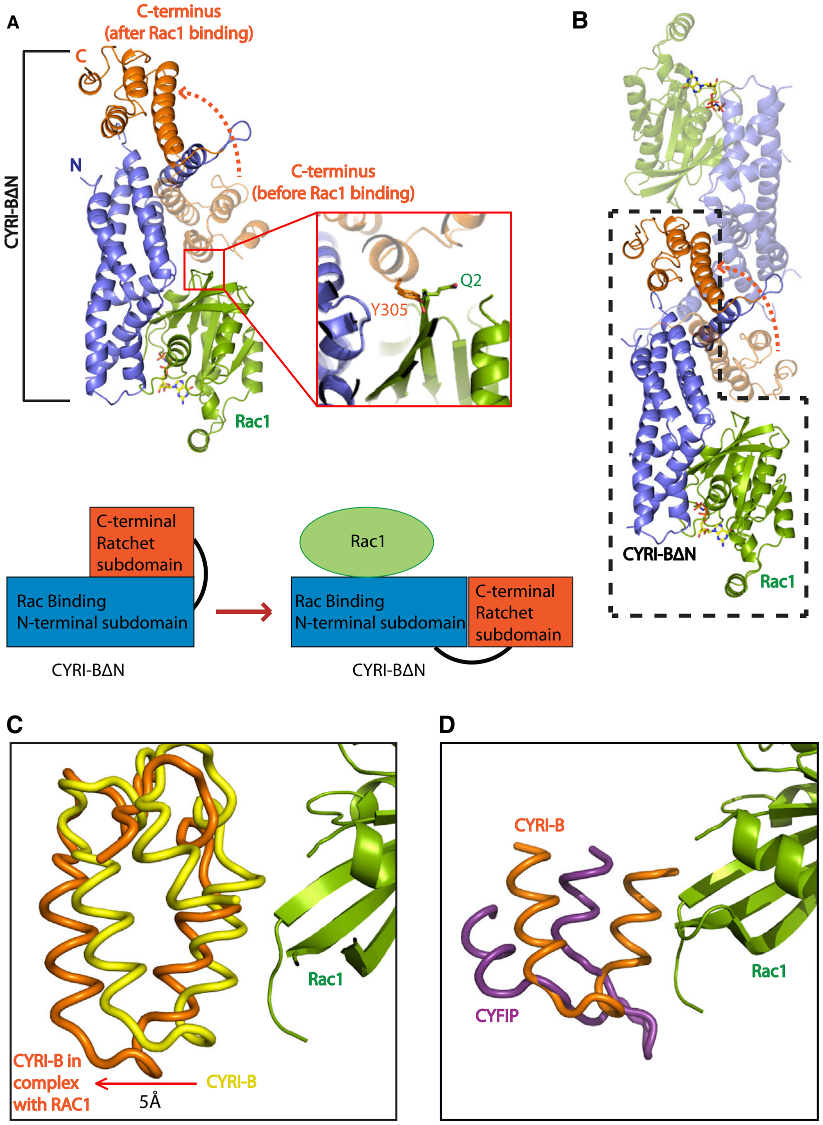

Figure 5. Conformational Changes in CYRI-BΔN Induced by Rac1Q61L Binding

(A) Crystal structure of CYRI-BΔN:Rac1Q61L shows a domain-swapped dimer. Overlay of CYRI-BΔN:Rac1Q61L complex with CYRI-BΔN Ratchet subdomain shown at 50% transparency. Rac1 shown in green with bound GppNHp in yellow stick form. The N-terminal helical bundle (residues 26–204) of CYRI-BΔN is shown in blue and the C-terminal helical bundle (residues 205–324) are shown in orange. The zoom-in box (in red) highlights the steric clashes between Y305 of CYRI-BΔN and Q2 of Rac1Q61L. The dotted orange arrow highlights the conformational change of the Rachet subdomain of CYRI-BΔN induced by Rac1 binding.

Schematic of subdomain organization of CYRI-BΔN shown highlighting the Rac1 binding and Ratchet subdomains and conformational changes induced through Rac1 binding.

(B) Domain swap induced by Rac1 binding. Overlaying the CYRI-B dimer with one dimer mate shown in yellow surface representation at 50% transparency proposes a swapping of the Rachet subdomain between CYRI monomers. Rac1 shown in green with the bound nucleotide in stick form.

(C) The 5Å shift of the C-terminal helical bundle between CYRI-BΔN structure(yellow) with CYRI-BΔN:Rac1Q61L complex (orange) allows for Rac1Q61L (green) binding.

(D) Comparison of CYRI-BΔN (orange) with CYFIP PDB: 3P9C (purple) shows that the steric clash is unique to CYRI-B. Rac1Q61L shown in green.

template to model this interaction. Overlaying of the CYRI-BΔN:Rac1Q61L structure with the Scar/WAVE complex (PDB: 3P8C) shows that Rac1 contacts the “A” site (residues on CYFIP adjacent to α4-α6 on the meander region of WAVE1) as previously proposed (Chen et al., 2010) (Figures 7A and 7B). All CYFIP residues identified as involved in Rac1 binding are at the docked interface. Of the residues reported (R190, C179, E434, F626, and M632), only R161 (R190 in CYFIP) is conserved with CYRI. We, therefore, set out to identify a minimal conserved DUF1394 domain Rac1 binding interface, using a sequence alignment of CYFIP1/2 and CYRI-A/B coupled with our CYRI-BΔN:Rac1Q61L complex (Figures 6A, S5A, and S5B). Despite the low sequence identity, nine residues (Q68, N154, S157, R160, R161, N185, S188, L189 and A192) at the interface are either conserved or have similar chemical properties. This supports that the binding mechanism is conserved between CYRI and CYFIP allowing for a more informed model of how Rac1 interacts with the Scar/WAVE complex.

A previous model proposed that Rac1 would either interact directly with the meander region of WAVE1 or Rac1 binding would serve to alter the meander regions stability – allowing for the VCA region to become Arp2/3 binding competent (Chen et al., 2010). Using our model, we observe no direct interaction between Rac1 and WAVE1. Instead, a steric clash is observed between the switch-I loop of Rac1 and a loop on CYFIP, which is adjacent to the α4 helix of WAVE (Figure 7C). We propose that binding of Rac1 would induce a conformational change in CYFIP, mediated through steric clashes with Rac1’s switch-I, which would destabilize the meander region of WAVE and a structural rearrangement in the adjacent VCA helices to facilitate the binding and activation of the Arp2/3 complex.

Phosphorylation of Y151 in WAVE has also been implicated in Scar/WAVE activity (Stuart et al., 2006). Y151 is buried and therefore unlikely to be phosphorylated without a necessary conformational change. In our model of Rac1 binding, Rac1 would induce conformational changes in CYFIP residues that bind to Y151 of WAVE. It is therefore feasible that these conformational changes would allow Y151 to be exposed for its phosphorylation, to enhance Scar/WAVE activity.

In summary, the Rac1 binding interface is evolutionarily conserved among DUF1394-containing proteins. This conservation

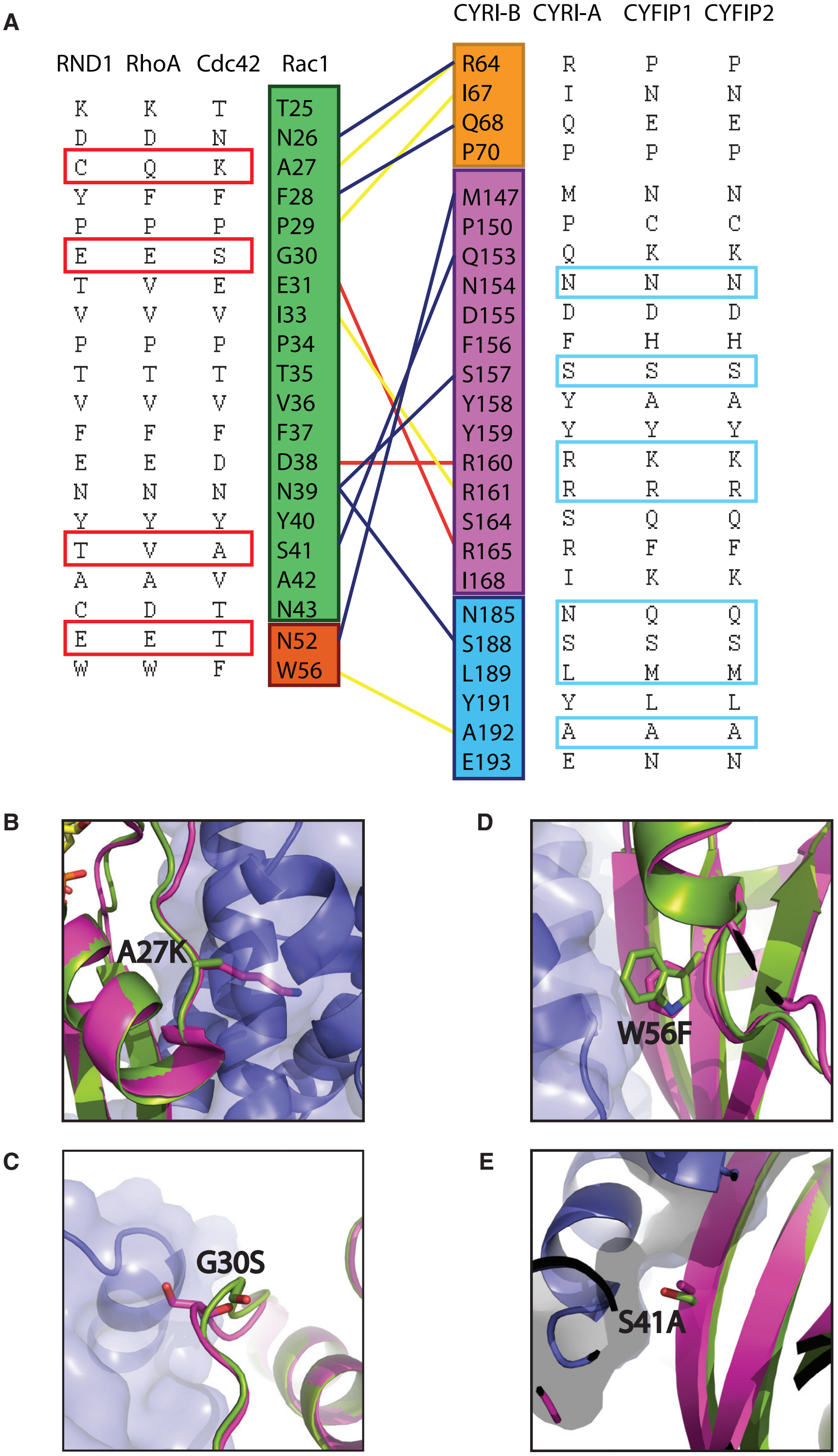

Figure 6. Structural Basis for Rac1 Selectivity to CYRI

(A) Sequence comparison of four members of the Rho GTPase family, RND1, RhoA, Cdc42, and Rac1. Red boxes highlight residues that are different between Rac1 and the others, which likely contribute to selectivity. Sequence comparison of human DUF1394 domains from CYRI-A, CYRI-B, CYFIP-1, and CYFIP-2. Residues that are conserved or with similar biochemical properties at the Rac1 binding interface are highlighted in blue boxes.

(B–E) Structural comparison of Rac1 (green) to Cdc42 PDB: 4JS0 (pink) highlighting residues involved in selectivity for CYRI-B (blue).

allows for the docking of Rac1 onto the "A" site of the Scar/WAVE complex (Chen et al., 2017). Our model provides a starting point for further examination of functionally important residues to further elucidate the activation mechanisms of the Scar/WAVE complex.

DISCUSSION

Actin polymerization provides a driving force for membrane protrusion during lamellipodia extension and migration. It is increasingly clear that positive and negative feedback loops control actin dynamics and allow cells to respond quickly to environmental cues such as chemoattractants, adhesion molecules, or changes in rigidity. The Scar/WAVE complex is the major controller of actin in lamellipodia downstream of Rac1 and was recently shown to be negatively regulated by CYRI proteins (Fort et al., 2018). Understanding how positive and negative signals influence actin dynamics is crucial to our understanding of cell migration, developmental processes, and pathological conditions such as cancer. While the interaction between Rac1 and the Scar/WAVE complex has been modeled based on their individual 3D structures (Chen et al., 2017), we here elucidate the structural interface between Rac1 and CYRI proteins, which serve as a structural analogue of the CYFIP:Rac1 binding "A" site.

Here, we report the novel crystal structures of the CYRI-BΔN alone and in complex with Rac1. CYRI-B binds Rac1 through a unique binding interface using extensive contacts with the switch-I loop and is distinct from other Rac1 effector complexes. Using this structure and sequence alignments, we provide a molecular basis for DUF1394 domain selectivity toward Rac1 over other members of the Rho GTPase family. Our study reveals how Rac1 interacts with the DUF1394 and the conservation between CYFIP and CYRI proteins, albeit with some important differences that impact on the likely mechanism of their competition for active Rac1.

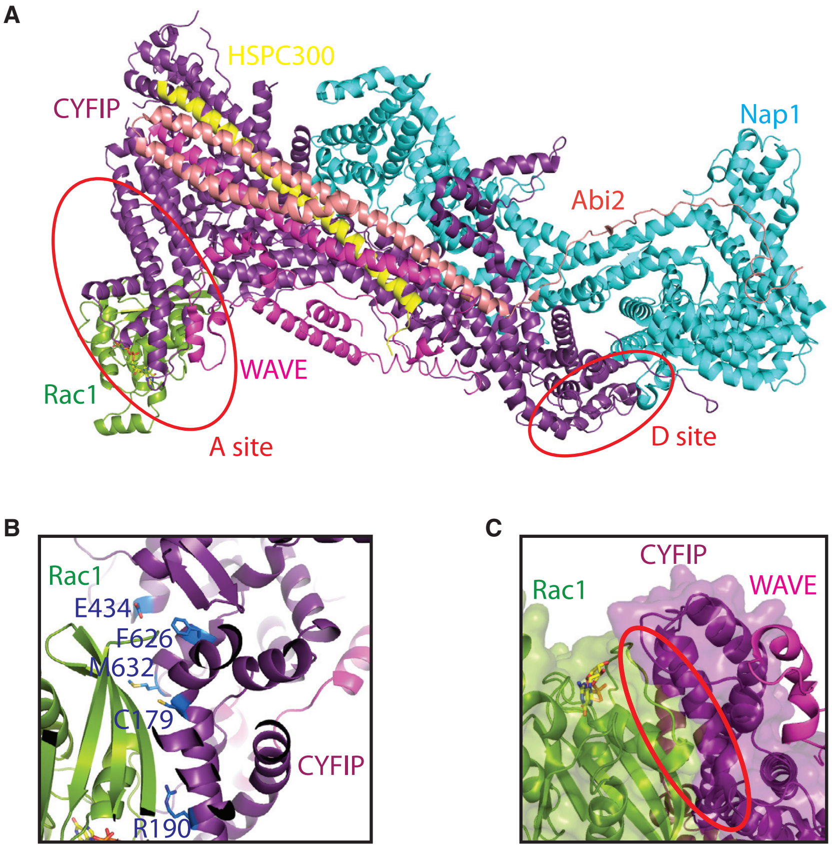

Figure 7. Docking of Rac1 onto the Scar/WAVE Complex

(A) Using our CYRI-BΔN:Rac1Q61L complex, Rac1 is docked onto the Scar/WAVE complex (PDB: 3P8C). Rac1Q61L binds to the "A" site of CYFIP adjacent to the meander region of WAVE. The "A" and "D" sites are highlighted in red circles.

(B) Mutations on CYFIP identified by Chen et al. (2010) that alter Rac1 binding are highlighted in blue stick form and are located at the interface between CYFIP and docked Rac1.

(C) A steric clash (red circle) occurs with switch one of Rac1 and a loop on CYFIP adjacent to the meander region of WAVE, providing a basis for conformational changes required for Scar/WAVE activation.

The crystal structure of CYRI-BΔN reveals the presence of an autoinhibited dimer present in solution and in cells as revealed through pulldowns and PLA. The dimerization of CYRI proteins could provide a basis for cooperative inhibition of lamellipodia protrusions in keeping with the known ability of CYRI to sharpen and increase lamellipodia dynamics (Fort et al., 2018; Linsay et al., 2019). Removal of autoinhibition will increase the affinity of CYRI to Rac1. Whether specific post-translational modifications or a membrane environment is required to regulate this autoinhibition is an intriguing possibility. Alternatively, CYRI association with the Scar/WAVE complex could allow specific regulation beyond sequestration of Rac1 proteins. Indeed, if autoinhibitory dimers are a conserved feature of DUF1394 domains, the possibility that Scar/WAVE could also form autoinhibited dimers and that CYRI could disrupt such interactions cannot be excluded. In fact, there have been several reports suggesting that the Scar/WAVE complex could form oligomeric structures (Pipathsouk et al., 2019), making this an attractive question for further investigation.

CYRI-B, unlike CYFIP or the Scar/WAVE complex, is myristoylated (Fort et al., 2018) and is thought to be transiently membrane-associated (Model, Figure S6). CYFIP and the Scar/WAVE complex depend on their interactions with the membrane-bound active Rac1 and acidic phospholipids to promote its own membrane association in "permissive zones" where these favorable conditions exist (Model, Figure S6). It would be interesting to study further the biological function of the Rachet subdomain, and whether its dynamics affect CYRI-B activity. It is conceivable that ensembles of CYRI are present in solution, where an open or a closed conformation can be stabilized by Rac1 binding or dimerization. This could allow CYRI-B to be regulated and switched on/off in response to Rac1 binding. Nevertheless, the existence of such a Ratchet subdomain and the implications of the conformational change suggest further tests of biological function to reveal how the Rac1 – CYRI-B – Scar/WAVE complex feedback loop is controlled are necessary.

Since our crystal structure revealed a similar fold between CYRI and CYFIP DUF1394, we modeled Rac1 binding to the CYFIP "A" site. Due to the sequence conservation, overlaying of our complex structure with that of the Scar/WAVE complex (Chen et al., 2010) reveals the likely interface between Rac1 and CYFIP at the "A" site. Strikingly, the structure reveals that there is no basis for the previously suggested direct competition between Rac1 and the meander region of CYFIP (Chen et al., 2010). Instead, we observe a steric clash between switch-I of Rac1 and CYFIP and we propose this as the driving force behind the release of the VCA region of WAVE. The caveat remains that the crystal structure of Scar/WAVE does not contain the proline-rich (residues 187–484) region of WAVE, and direct competition with these residues cannot be ruled out. Nevertheless, our model provides the basis for further experimentation and brings us closer to understanding Rac1 driven activation of the Scar/WAVE complex.

In conclusion, our structures of CYRI-BΔN and CYRI-BΔN:Rac1Q61L reveal the nature of the inhibition of Rac1-mediated activation of the Scar/WAVE complex by CYRI proteins and affirm the DUF1394 domain as an evolutionarily conserved Rac1 binding module. We reveal how Rac1 binding can drive a dramatic conformational change in CYRI, provide a basis for autoinhibited dimerization and the ability of CYRI to compete with the Scar/WAVE complex for Rac1 binding. Docking of our complex onto the Scar/WAVE complex additionally provides structural evidence that current models for how Rac1 activates Scar/WAVE complex will need to be revised.

STAR★METHODS

Detailed methods are provided in the online version of this paper and include the following:

- KEY RESOURCES TABLE
- RESOURCE AVAILABILITY
  - Lead Contact
  - Materials Availability
  - Data and Code Availability
- EXPERIMENTAL MODEL AND SUBJECT DETAILS
- METHOD DETAILS
  - Construct Design
  - Protein Expression and Purification
  - Crystallisation of CYRI-BΔN and CYRI-BΔN-Rac1Q61L.GppNHp
  - Structure Determination
  - Antibodies
  - MBP Pulldown Assay
  - Proximity Ligation Assay
  - Surface Plasmon Resonance Assay
- QUANTIFICATION AND STATISTICAL ANALYSIS

SUPPLEMENTAL INFORMATION

Supplemental Information can be found online at https://doi.org/10.1016/j.str.2020.11.003.

ACKNOWLEDGMENTS

This work was supported by Cancer Research UK core funding number A17196. We thank the Core Services and Advanced Technologies at the Cancer Research UK Beatson Institute with particular thanks to Beatson Advanced Imaging Resource, Biological Services Unit and Molecular Technologies. We thank Diamond Light Source beamlines I03 and I04 for help with data collection.

AUTHOR CONTRIBUTIONS

TY obtained and solved crystal structures. AHL performed pulldown and PLA experiments. SN performed cloning. SI, RI, and LM conceived the project. TY, AHL, LM, and SI wrote the paper.

DECLARATION OF INTERESTS

The authors declare that they have no conflict of interest.

Received: June 30, 2020
Revised: October 7, 2020
Accepted: November 2, 2020
Published: November 19, 2020

REFERENCES

Chen, B., Chou, H.T., Brautigam, C.A., Xing, W., Yang, S., Henry, L., Doolittle, L.K., Walz, T., and Rosen, M.K. (2017). Rac1 GTPase activates the WAVE regulatory complex through two distinct binding sites. Elife 6, e29795.

Chen, Z., Borek, D., Padrick, S.B., Gomez, T.S., Metlagel, Z., Ismail, A.M., Umetani, J., Billadeau, D.D., Otwinowski, Z., and Rosen, M.K. (2010). Structure and control of the actin regulatory WAVE complex. Nature *468*, 533–538.

Davidson, A., and Insall, R. (2011). Actin-based motility: WAVE regulatory complex structure reopens old SCARs. Curr. Biol. *21*, R66–R68.

Davis, M.J., Ha, B.H., Holman, E.C., Halaban, R., Schlessinger, J., and Boggon, T.J. (2012). RAC1P29S is a spontaneously activating cancer-associated GTPase. Proc. Natl. Acad. Sci. U S A *110*, 912–917.

Davis, M.J., Ha, B.H., Holman, E.C., Halaban, R., Schlessinger, J., and Boggon, T.J. (2013). RAC1P29S is a spontaneously activating cancer-associated GTPase. Proc. Natl. Acad. Sci. U S A *110*, 912–917.

Emsley, P., and Cowtan, K. (2004). Coot: model-building tools for molecular graphics. Acta Crystallogr. D Biol. Crystallogr. *60*, 2126–2132.

Emsley, P., Lohkamp, B., Scott, W.G., and Cowtan, K. (2010). Features and development of COOT. Acta Crystallogr. D Biol. Crystallogr. *66*, 486–501.

Fort, L., Batista, J.M., Thomason, P.A., Spence, H.J., Whitelaw, J.A., Tweedy, L., Greaves, J., Martin, K.J., Anderson, K.I., Brown, P., et al. (2018). Fam49/CYRI interacts with Rac1 and locally suppresses protrusions. Nat. Cell Biol. *20*, 1159–1171.

Hodis, E., Watson, I.R., Kryukov, G.V., Arold, S.T., Imielinski, M., Theurillat, J.P., Nickerson, E., Auclair, D., Li, L., Place, C., et al. (2012). A landscape of driver mutations in melanoma. Cell *150*, 251–263.

Hoeller, O., Toettcher, J.E., Cai, H., Sun, Y., Huang, H., Freyre, M., Zhao, M., Devreotes, P.N., and Weiner, O.D. (2016). Gbeta regulates coupling between actin oscillators for cell polarity and directional migration. PLoS Biol. *14*, e1002381.

Holm, L. (2020). DALI and the persistence of protein shape. Protein Sci. *29*, 128–140.

Kast, D.J., Yang, C., Disanza, A., Boczkowska, M., Madasu, Y., Scita, G., Svitkina, T., and Dominguez, R. (2014). Mechanism of IRSp53 inhibition and combinatorial activation by Cdc42 and downstream effectors. Nat. Struct. Mol. Biol. *21*, 413–422.

Krissinel, E., and Henrick, K. (2007). Inference of macromolecular assemblies from crystalline state. J. Mol. Biol. *372*, 774–797.

Krause, M., and Gautreau, A. (2014). Steering cell migration: lamellipodium dynamics and the regulation of directional persistence. Nat. Rev. Mol. Cell Biol. *15*, 577–590.

Krauthammer, M., Kong, Y., Ha, B.H., Evans, P., Bacchiocchi, A., McCusker, J.P., Cheng, E., Davis, M.J., Goh, G., Choi, M., et al. (2012). Exome sequencing identifies recurrent somatic RAC1 mutations in melanoma. Nat. Genet. *44*, 1006–1014.

Kühn, S., Erdmann, C., Kage, F., Block, J., Schwenkmezger, L., Steffen, A., Rottner, K., and Geyer, M. (2015). The structure of FMNL2-Cdc42 yields insights into the mechanism of lamellipodia and filopodia formation. Nat. Commun. *6*, 7088.

Lapouge, K., Smith, S., Walker, P.A., Gamblin, S.J., Smerdon, S.J., and Rittinger, K. (2000). Structure of the TPR domain of p67phox in complex with Rac.GTP. Mol. Cell *6*, 899–907.

Langer, G., Cohen, S.X., Lamzin, V.S., and Perrakis, A. (2008). Automated macromolecular model building for X-ray crystallography using ARP/wARP version 7. Nat. Protoc. *3*, 1171–1179.

Linsay, C., Xu, Z., Ditlev, J.A., and Rosen, M.K. (2019). Stoichiometry controls activity of phase-separated clusters of actin signaling proteins. Science *6431*, 1093–1097.

Modha, R., Campbell, L.J., Nietlispach, D., Buhecha, H.R., Owen, D., and Mott, H.R. (2008). The Rac1 polybasic region is required for interaction with its effector PRK1. J. Biol. Chem. *283*, 1492–1500.

Murshudov, G., Vagin, A., and Dodson, E. (1997a). Refinement of macromolecular structures by the maximum-likelihood method. Acta Crystallogr. D Biol. Crystallogr. *D53*, 240–255.

Murshudov, G., Alexei Vagin, A., and Dodson, E. (1997b). Refinement of macromolecular structures by the maximum-likelihood method. Acta Crystallogr. D Biol. Crystallogr. *D53*, 240–255.

Pipathsouk, A., Brunetti, R.M., Town, J.P., Breuer, A., Pellett, P.A., Marchuk, K., Tran, N.T., Krummel, M.F., Stamou, D., and Weiner, O.D. (2019). WAVE complex self-organization templates lamellipodial formation. bioRxiv. https://doi.org/10.1101/836585.

Schaks, M., Singh, S.P., Kage, F., Thomason, P., Klünemann, T., Steffen, A., Blankenfeldt, W., Stradal, T.E., Insall, R.H., and Rottner, K. (2018). Distinct

CellPress
OPEN ACCESS

interaction sites of rac GTPase with WAVE regulatory complex have non-redundant functions in vivo. Curr. Biol. *28*, 3674–3684 e3676.

Shang, W., Jiang, Y., Boettcher, M., Ding, K., Mollenauer, M., Liu, Z., Wen, X., Liu, C., Hao, P., Zhao, S., et al. (2018). Genome-wide CRISPR screen identifies FAM49B as a key regulator of actin dynamics and T cell activation. Proc. Natl. Acad. Sci. U S A *115*, E4051–E4060.

Stuart, J.R., Gonzalez, F.H., Kawai, H., and Yuan, Z.-M. (2006). c-Abl interacts with the WAVE2 signaling complex to induce membrane ruffling and cell spreading. J. Biol. Chem. *281*, 31290–31297.

Tarricone, C., Xiao, B., Justin, N.N., Walker, P.A., Rittinger, K., Gamblin, S.J., and Smerdon, S.J. (2001). The structural basis of Arfaptin-mediated cross-talk between Rac and Arf signalling pathways. Nature *411*, 215–219.

Veltman, D., King, J.S., Machesky, L.M., and Insall, R.H. (2012). SCAR knock-outs in Dictyostelium: WASP assumes SCAR's position and upstream regulators in pseudopods. J. Cell Biol *198*, 501–508.

Winn, M.D., Ballard, C.C., Cowtan, K.D., Dodson, E.J., Emsley, P., Evans, P.R., Keegan, R.M., Krissinel, E.B., Leslie, A.G., McCoy, A., et al. (2011). Overview of the CCP4 suite and current developments. Acta Crystallogr. D Biol. Crystallogr. *67*, 235–242.

Winter, G. (2010). *xia2:* an expert system for macromolecular crystallography data reduction. J. Appl. Crystallogr. *43*, 186–190.

Whitelaw, J.A., Lilla, S., Paul, N.R., Fort, L., Zanivan, S., and Machesky, L.M. (2019). CYRI/Fam49 proteins represent a new class of Rac1 interactors. Communicative Integr. Biol. *12*, 112–118.

Yuki, K.E., Marei, H., Fiskin, E., Eva, M.M., Gopal, A.A., Schwartzentruber, J.A., Majewski, J., Cellier, M., Mandl, J.N., Vidal, S.M., et al. (2019). CYRI/FAM49B negatively regulates RAC1-driven cytoskeletal remodelling and protects against bacterial infection. Nat. Microbiol. *4*, 1516–1531.

STAR★METHODS

KEY RESOURCES TABLE

REAGENT or RESOURCE	SOURCE	IDENTIFIER
**Antibodies**		
mouse monoclonal anti-FLAG M2	Sigma-Aldrich	Cat: F3165; RRID: AB_259529
rabbit monoclonal anti-HA tag	Cell Signalling Technology	Cat: 3724; RRID: AB_1549585
Mouse monoclonal anti-MBP	New England Biolabs	Cat: 8032S; RRID: AB_1559730
Rabbit anti-RAC1/2/3	Cell Signalling Technology	Cat: 2465; RRID: AB_2176152
Rabbit anti-GST	Cell Signalling Technology	Cat: 2622; RRID: AB_331670
**Bacterial and Virus Strains**		
E. coli BL21(DE3) – Novagen	Sigma-Aldrich/ Merck	Cat: 69450-3
E. coli B834(DE3) – Novagen	Sigma-Aldrich/ Merck	Cat: 69041-3
E. coli DH5a	Homemade	N/A
**Chemicals, Peptides, and Recombinant Proteins**		
Restriction endonucleases: BamHI, HindIII, EcoRI, NotI	New England Biolabs	BamHI: R0136S, HindIII: R0104S, EcoRI: R0101S, NotI: R3189S
Ampicillin sodium salt	Formedium	AMP100
Kanamycin	Formedium	KNM0100
IPTG	Formedium	IPTG250
Selenomethionine media	Molecular Dimensions	MD12-500
GppNHp	JenaBioscience	NU-899-50
Q5 Site directed mutagenesis kit	New England Biolabs	E0554S
**Critical Commercial Assays**		
Duolink In Situ Red Starter Kit Mouse/ Rabbit	Sigma-Aldrich	DUO92101
Duolink In Situ PLA Probe Anti-Mouse PLUS	Sigma-Aldrich	DUO92001
Duolink In Situ PLA Probe Anti-Rabbit PLUS	Sigma-Aldrich	DUO92002
**Deposited Data**		
Rac1 Q61L	(Davis et al., 2013)	PDB: 4GZL
CYRI-BΔN	This work	PDB: 7AJL
CYRI-BΔN-Rac1 Q61L Fusion	This work	PDB: 7AJK
Scar/WAVE complex	Chen et al. (2010)	PDB: 3P8C
Cdc42	Kast et al. (2014)	PDB: 4JS0
Arfaptin	Tarricone et al. (2001)	PDB:1I4T
p67Phox	Lapouge et al. (2000)	PDB: 1E96
PRK	Modha et al. (2008)	PDB: 2RMK
**Experimental Models: Cell Lines**		
COS-7	ATCC	CRL-1651
**Oligonucleotides**		
All oligomers are provided in Table S2.	This work	N/A
**Recombinant DNA**		
Codon optimized Human CYRI-BΔN Rac1 Q61L Fusion Cloned into MBP pRSF Duet	This work/IDT	N/A
MBP Mouse CYRI-B R160D cloned into pMAL-C5X	This work/IDT	N/A
MBP Mouse CYRI-B R161D cloned into pMAL-C5X	This work/IDT	N/A

(*Continued on next page*)

CellPress
OPEN ACCESS

Continued

REAGENT or RESOURCE	SOURCE	IDENTIFIER
MBP-HA tagged Mouse CYRI-B cloned into MBP pRSF Duet	This work	N/A
Human Rac1Q61L S41A cloned into pGEX-2T1	This work	N/A
Human Rac1Q61L S157A cloned into pGEX-2T1	This work	N/A
Human Rac1Q61L S157R cloned into pGEX-2T1	This work	N/A
Human Rac1Q61L Q153R cloned into pGEX-2T1	This work	N/A
Human Rac1Q61L cloned into pGEX-2T1	This work	N/A
Software and Algorithms		
CCP4 (including MOLREP, and REFMAC5)	(Winn et al., 2011)	http://www.ccp4.ac.uk/
COOT	(Emsley et al., 2010)	https://www2.mrc-lmb.cam.ac.uk/personal/pemsley/Coot/
PISA	(Krissinel and Henrick, 2007)	https://www.ebi.ac.uk/pdbe/pisa/
PyMOL	Schodinger, LLC	https://pymol.org/2/
Xia2 (including XDS and SCALA)	(Winter, 2010)	https://xia2.github.io/index.html
Prism8	GraphPad	https://www.graphpad.com/scientific-software/prism/
DALI Server	(Holm, 2020)	http://ekhidna2.biocenter.helsinki.fi/dali/
Other		
HisTrap HP	Cytivia	Cat: 17524701
MBPTrap	Merch	Cat: GE28-9187-79
GSTTrap	Cytivia	Cat: GE17-5131-01
MBP-Trap Agarose	ChromoTek	Cat: mbta-20
Superdex S75 16/60	Cytivia	Cat: 28989333
Superdex S200 16/60	Cytivia	Cat: 28989335
Biacore T200	Cytivia	Cat: 28975001
Series S CM5 Chip	Cytivia	Cat: 29104988

RESOURCE AVAILABILITY

Lead Contact

Further information and requests for resources and reagents should be directed to and will be fulfilled by the Lead Contact Shehab Ismail, email: Shehab.Ismail@glasgow.ac.uk.

Materials Availability

All the materials generated in this study are available from the Lead Contact upon request.

Data and Code Availability

Protein structures were deposited in the PDB accession codes: PDB: 7AJL for CYRI-BΔN and PDB: 7AJK for CYRI-BΔN:Rac1Q61L.

EXPERIMENTAL MODEL AND SUBJECT DETAILS

Unlabelled protein was expressed and purified from *Escherichia coli* BL21 (DE3) cells grown in LB media at 37°C until OD_{600} reached ~0.6. Cells were cooled to 20°C and induced with 0.2mM IPTG and left to express for 20 hours. Selenomethionine labelled protein was expressed using B834 (DE3). Cells were grown in minimal media at 37°C until OD_{600} reached ~0.6. Cells were cooled to 20°C washed in PBS and added to selenomethionine labelled minimal media (Molecular Dimensions) and left to express overnight.COS7 cells (Cercopithecus aethiops-ATCC) are cultured using standard culturing method with DMEM (Gibco) supplemented with 10% serum, 1X glutamine and 1X Penicillin/Streptomycin. Cells are maintained at 37°C at 5% CO2 and are passaged every Monday and Friday. Cells are used for experiments no longer than P20.

METHOD DETAILS

Construct Design

All GST-tagged Rac1Q61L and its mutants were cloned into pGEX2T backbone, while CYRI-B and its mutants used for pulldowns were cloned into pMAL-C5X vector (NEB). MBP CYRI-BΔN and MBP CYRI-BΔN:Rac1Q61L fusion were cloned into pRSF_Duet cloned to have a His_{10} tagged MBP that was TEV cleavable at the N-terminus.

Mutagenesis was done using the Q5 Site-directed Mutagenesis kit (NEB, #E0554) according to the manufacturer protocol. Primers were designed using NEB BaseChanger website. The human CYRI-BΔN: Rac1 fusion was synthesised as a codon optimised gBlock (Integrated DNA Technologies) for expression in *E. coli*. The construct contained human CYRI-B residues 26-324, a ten-residue linker GSAGSAGSAG and human Rac1Q61L residues 2-177.

Protein Expression and Purification

All GST fusion constructs were grown in the presence of $100ugml^{-1}$ ampicillin, all cleavable-MBP fusion constructs used for crystallography were grown in the presence of $50ugml^{-1}$ kanamycin, whilst all MBP fusion constructs for pulldown assays were grown in the presence of $50ugml^{-1}$ ampicillin. All constructs were expressed in BL21 pLysS (Promega). Cells were grown at 37°C until OD_{600} reached 0.4-0.6 and induced with 0.4mM IPTG and left to express at 20°C for 16 hours.

Purification of GST-Rac1

Cells were lysed using a microfluidizer at 20,000psi in a buffer containing 20mM Tris pH 8.0, 300mM NaCl, 5mM $MgCl_2$ and 2mM betamercaptoethanol (BME). Cell lysates were clarified by centrifugation followed by filtration through a 0.45μm filter. Clarified lysate was loaded onto a GSTrap column (GE Healthcare). Once loaded, the column was washed with 50ml of lysis buffer before adding thrombin (Sigma) and cleaving on the column overnight at 16°C. The next day the protein was pooled, concentrated and passed over an S75 size exclusion column equilibrated in 20mM Tris, 150mM NaCl, 5mM $MgCl_2$ and 2mM DTT.

Expression and Purification of MBP-CYRI-B⊿N for Seleno-Methionine

To ensure efficient incorporation of seleno-methionine into the expressed protein, B834 (DE3) cells (Novagen) were used. An overnight culture was grown at 37°C in LB. Unlabelled media (Molecular Dimesions) was inoculated with 10ml of overnight culture and grown until OD_{600} reached 0.5. Cells were harvested and washed three times in PBS before resuspending the pellets in seleno-methionine labelled media (Molecular Dimension). After a 40 minute incubation at 20°C, cells were induced with 0.4mM IPTG and left to express for 16 hours.

Harvested cells were lysed using a microfluidizer at 20,000 psi in a buffer containing 50mM Tris pH 8.0, 300mM NaCl, and 5mM BME. Cell lysates were clarified by centrifugation at 20,000xg and filtered through a 0.45μm filter. Lysates were then loaded onto a HisTrap column (GE Healthcare) before washing with 20mM imidazole. The protein was eluted with a linear gradient of imidazole from 0-300mM. Fractions containing protein were pooled and dialysed overnight at 4°C in the presence of TEV in a buffer containing 20mM Tris pH 8.0, 150mM NaCl, 5mM imidazole, and 2mM BME. The following day, proteins were passed over a HisTrap column and the flowthrough collected which was concentrated and passed over a Superdex S200 (GE Healthcare) column equilibrated in 10mM Tris pH7.5, 50mM NaCl and 2mM DTT.

Purification of MBP-CYRI-B⊿N-Rac1Q61L Fusion and HA-CYRI-B Constructs

Harvested cells were lysed using a microfluidizer at 20,000 psi in a buffer containing 50mM Tris pH 8.0, 300mM NaCl, 5mM $MgCl_2$ and 5mM BME. Cell lysates were clarified by centrifugation at 20,000xg and filtered through a 0.45μm filter. Lysates were then loaded onto a HisTrap column (GE Healthcare) before washing with 20mM imidazole. The protein was eluted with a linear gradient of imidazole from 0-300mM. Fractions containing protein were pooled and dialysed overnight at 4°C in the presence of TEV in a buffer containing 20mM Tris pH 8.0, 150mM NaCl, 5mM imidazole, 5mM $MgCl_2$ and 2mM BME. The following day, proteins were passed over a HisTrap column and the flowthrough collected and concentrated to 1ml. Nucleotide exchange was performed for 2 hours by adding EDTA to a final concentration of 50mM and GppNHp at a 10:1 molar ratio. Finally, the protein was passed over an S200 size exclusion column (GE Healthcare) equilibrated in a buffer containing 10mM Tris pH 8.0, 50mM NaCl, 4mM $MgCl_2$ and 5mM DTT.

Small-Scale Purification of GST-tagged and MBP-tagged Proteins for Pulldown Assay

Day 1, 200ml of transformed BL21 competent bacteria is grown overnight at 37°C in LB with the appropriate antibiotics. Day 2, 10ml of the preculture is transferred to 1L of LB and grow until the OD reaches 0.4. The bacteria are then induced with 0.2mM of IPTG overnight at room temperature. Day 3, the bacteria are then centrifuged at 3000rpm for 10min, then lysed in Buffer A (50mM Tris 7.5, 50mM NaCl, 5mM $MgCl_2$) containing 0.25mM DTT using sonication. The lysate is cleared by spinning at 20,000rpm for 20min and then incubated with pre-equilibrate GST beads (Glutathione Sepharose 4B #GE 17-0756-01) for 1h at 4°C. The GST-tagged protein is then eluted by incubating with Buffer A supplemented with 0.1% Tx100 and 10mM Glutathione. For MBP-tagged proteins, instead of using Buffer A as above, we use MBP Buffer (100mM NaCl, 10mM Tris pH7.5). Once the protein is conjugated to MBP beads, the beads can be kept at 4°C in the MBP Buffer until use.

The proteins are checked with Coomassie for purity and correct molecular weight.

Crystallisation of CYRI-BΔN and CYRI-BΔN-Rac1Q61L.GppNHp

Following a large sparse matrix crystallisation screen, initial rectangular crystals of CYRI-BΔN were obtained in Morpheus G8 at 291K at $8mgml^{-1}$. Crystals were optimised using reagents purchased from Molecular Dimension and contained 0.1M carboxylic acid, 8% MPD_P1K_P33 and 0.1M MOPS/HEPES-Na pH 8.0. Crystals were cryoprotected in the same condition with 20% MPD_P1K_P33 and flash-frozen in liquid nitrogen.

Crystals of CYRI-BΔN-Rac1Q61L were obtained in PEGsII C6 at 11mgml^{-1} and were optimised to contain 8% v/v PEG4,000, 0.1M Tris pH 8.5 and 0.2M Sodium acetate at 279K. Crystals were then flash-frozen in liquid nitrogen in a cryoprotectant containing the reservoir solution supplemented with 25% v/v glycerol.

Structure Determination

Data for CYRI-BΔN was obtained at Diamond Light Source beamline I04. Data were collected at the selenium absorption edge and the structure solved using single-wavelength anomalous strategy. Initial data processing was performed using BIG-EPS before completing an initial model using Arp/Warp (Langer et al., 2008). Final rounds of refinement and manual model building were performed using COOT (Emsley and Cowtan, 2004) and REFMAC5 (Murshudov et al., 1997a) of the CCP4 program suite.

Data for CYRI-BΔN:Rac1Q61L were obtained at Diamond Light Source beamline I03 and processed using Xia1. Initial phases were obtained using Molrep (Murshudov et al., 1997b) with the CYRI-BΔN and Rac1Q61L (PDB: 4GZL) structures as search models. Following initial phasing manual model building and ligand fitting (GppNHp and Mg^{2+}) was performed using COOT (Emsley and Cowtan, 2004) and REFMAC5 (Murshudov et al., 1997a) of the CCP4 program suite (Winn et al., 2011).

Antibodies

The following antibodies were used for western blot detection: Anti-MBP mouse monoclonal antibody (from NEB, #E8032S, 1:10,000 dilution), Anti-GST rabbit monoclonal antibody (from Cell Signalling Technology, #2625S, 1:1,000 dilution) anti-Rac1/2/3 rabbit antibody (Cell Signalling Technology, #2465). Antibodies used for Proximity Ligation Assay: mouse monoclonal anti-FLAG M2 (Sigma-Aldrich, #F3165, 1:800 dilution), rabbit monoclonal anti-HA tag (Cell Signalling Technology, #3724, 1:800 dilution). DAPI was used to stain for nucleic acid (Thermo Scientific, #62248, 1:1,000 dilution).

MBP Pulldown Assay

An equal amount of protein conjugated-MBP beads were used between conditions. For each reaction, 30μg of purified HA-tagged protein was mixed with the Binding Buffer (Buffer A pH 7.5 + 0.1% Tx100) for a total of 500μl and incubated with the MBP beads for 90min at 4°C. Once incubated, the beads were pelleted and washed 5 times with the Binding Buffer and subjected to a western blot. To each sample, 1X NuPAGE Reducing Agent (Invitrogen, #NP0004) and 1X NuPAGE LDS Sample Buffer (Invitrogen, #NP0007) were added and run on a precast NuPAGE 4-12% Bis-Tris Protein gel (Invitrogen, #NP0322BOX). MBP loading was checked using PonceuS staining and HA-tagged protein binding was detected using HA antibody.

MBP Pulldown Competition Assay

The same condition as in the normal MBP pulldown assay is used. However, in addition, we add an increased level of active Rac1 Q61L (0, 1, 5, 10 and 30μg of protein) to the final incubating volume of 500μl. A ponceuS was used to determine the loading control while anti-MBP, anti-HA and anti-Rac1/2/3 (#2465, CST) were used for immunoblotting.

Proximity Ligation Assay

COS-7 cells were co-transfected with CYRI-A-FLAG and P17-HA-CYRI-B constructs for 24h. The next day, cells are subjected to the assay according to the manufacturer's protocol (SigmaAldrich, #DUO92008). At least 10 random fields of view were imaged per coverslip.

Surface Plasmon Resonance Assay

All SPR experiments were performed on a Biocore T200 instrument. Anti-MBP antibody (NEB, #E8032S) was immobilised using amide coupling onto a CM5 SPR chip (GE Healthcare) following the manufacturer's protocol. MBP was passed through flow-cell 1 at 10μlmin^{-1} until 400 response units were registered. MBP-CYRI-BΔN was passed through flow-cell 2 at 10μlmin^{-1} until ~800 response units were immobilised. Rac1Q61L at concentrations 0.625μM-80μM were used. The resulting data was analysed using T200 data analysis software and graph generated using Prism8.

QUANTIFICATION AND STATISTICAL ANALYSIS

Statistics for crystal structures of CYRI-BΔN and CYRI-BΔN:Rac1 were determined using software listed in the Key Resources Table. Statistics generated from data processing and refinement are listed in Table1. SPR data was generated and analysed using Biacore analysis software and image generated using Prism7.Statistical analysis for point data can be found in figure legends, with n is the number of cells analysed or the number of western blots done. For all data, graph represents mean value with error bars are Standard Deviation of the Mean (SEM). For data that are more than 30 data points, no test was used for normality check. For western blot data, Shapiro-Wilk normality test is used prior to statistical analyses. To compare between more than 2 groups, an ANOVA with Dunnett's multiple comparisons test is used. All graphs and statistical analyses are done using Prism8.

Structure, Volume *29*

Supplemental Information

Structural Basis of CYRI-B Direct Competition with Scar/WAVE Complex for Rac1

Tamas Yelland, Anh Hoang Le, Savvas Nikolaou, Robert Insall, Laura Machesky, and Shehab Ismail

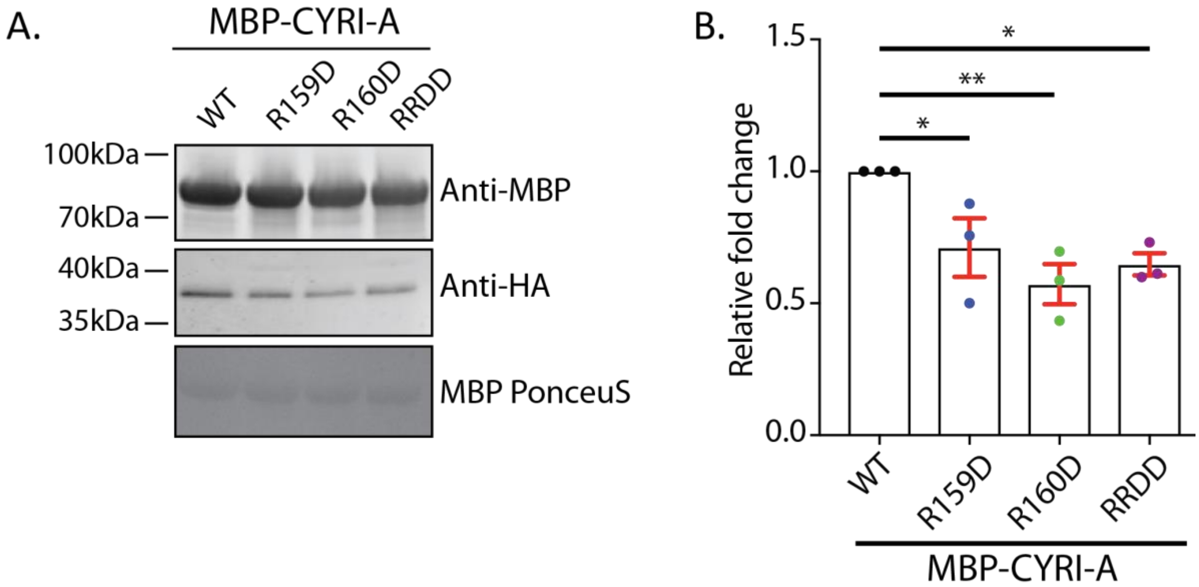

Supplementary Figure 1. Related to Figure 1. CYRI molecules can form heterotypic interactions and the conserved arginine residues play a role in this interaction.

A. Western blot analysis shows immobilised MBP-CYRI-A forms a weak but consistent interaction with HA-CYRI-B. Both single and double mutant of the conserved arginine residues can affect this heterodimerisation.
B. Quantification shows significant decreases in the dimerisation affinity between CYRI-A and CYRI-B when the single or double mutations were introduced.
Data from 3 independent experiments. Mean ± SEM. ANOVA with multiple comparisons. ns = $p>0.05$, *$p<0.05$, **$p<0.01$.

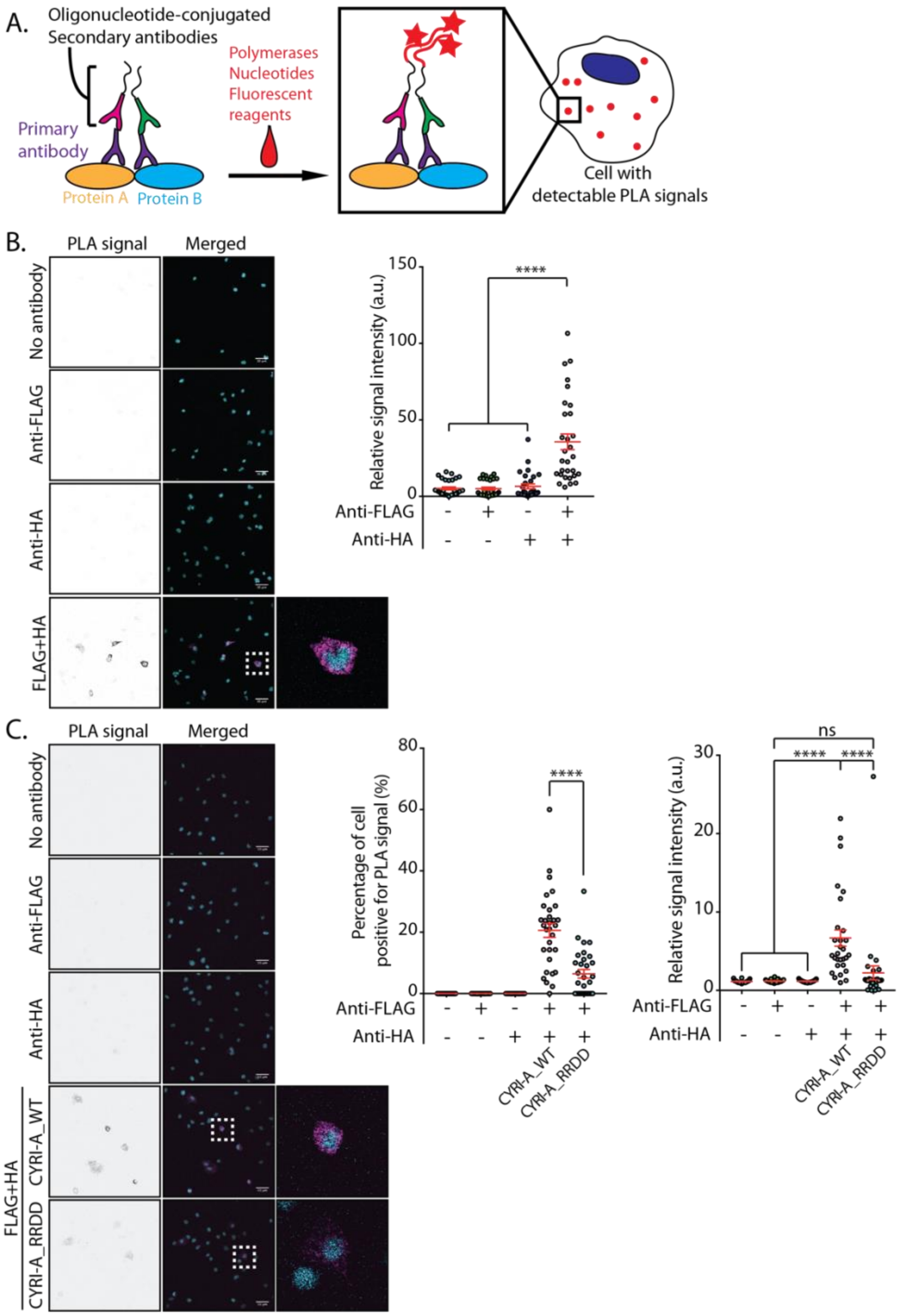

Supplementary Figure 2. Related to Figure 1. CYRI-A and CYRI-B interact with each other in vivo and the interaction is R159/R160 dependent.

A. Schematic representation of how the proximity ligation assay (PLA) works. Any protein within the 40nm proximity can generate the fluorescent PLA signal.
B. COS-7 cells were co-transfected with CYRI-A-FLAG and P17-HA-CYRI-B construct and proceed for a PLA. Only cells expressing both constructs and are probed with both anti-FLAG and anti-HA generated strong PLA signal.
C. Mutating both arginine 159 and 160 to aspartic acid in CYRI-A (RRDD), which are involved in active RAC1 binding, significantly decreases the PLA signal.
Scatter plots of 3 independent experiments. Mean ± SEM. ANOVA with multiple comparisons. ns = p>0.05, ****p<0.0001.

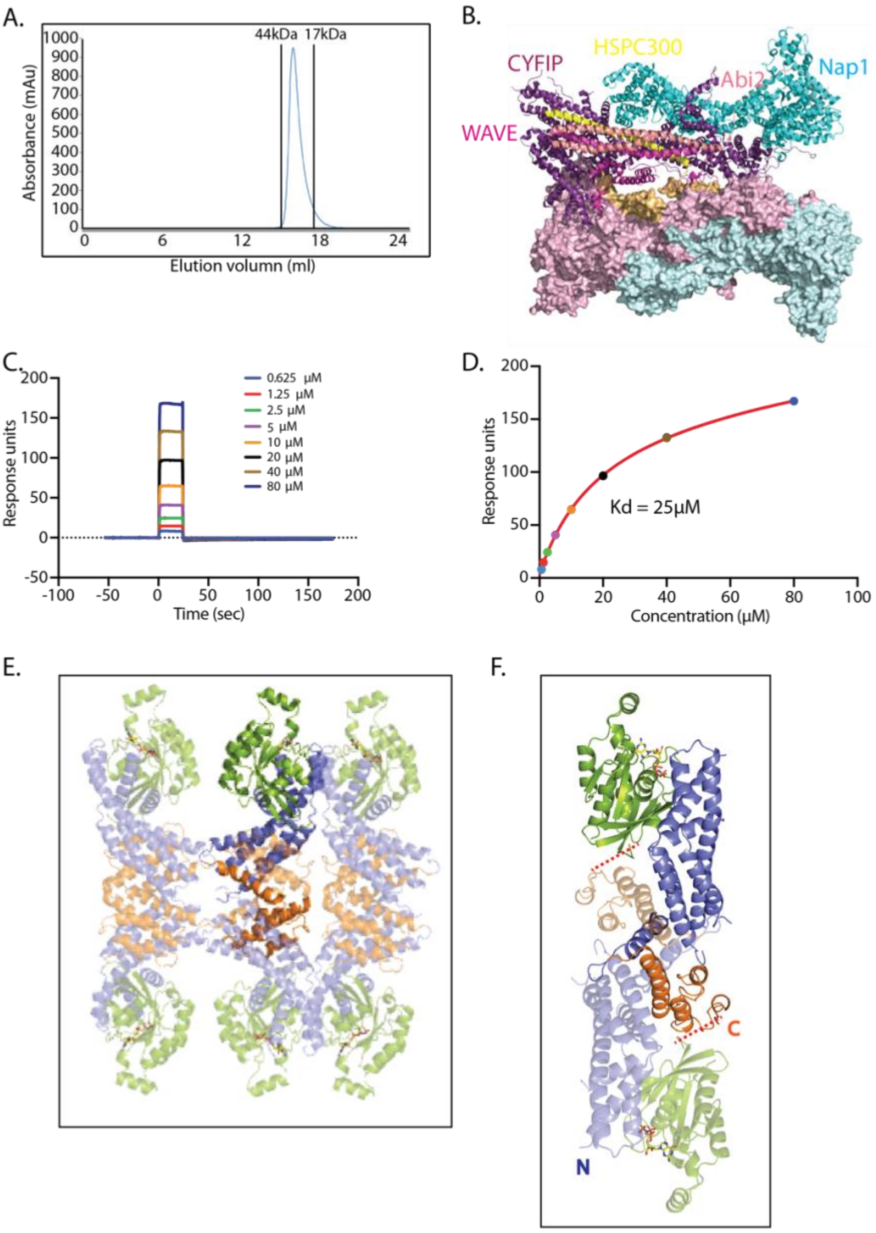

Supplementary Figure 3. Related to Figure 2, 3. CYRI-B oligomerisation, SPR binding curve for Rac1Q61L with MBP-CYRI-B and crystal packing of CYRI-BΔN:Rac1Q61L.

A. CYRI-B could not be detected through analytical size exclusion chromatography, potentially due to its weak affinity. Molecular weight marker elution volumes highlighted.
B. Theoretical Scar/WAVE complex dimers. One Scar/WAVE complex (PDB:3P8C) shown in cartoon form, the second in surface form.
C-D. SPR binding curve and affinity measurement using immobilised MBP-CYRI-B and titrating Rac1Q61L. Titration range from 0.625μM to 80μM Rac1Q61L. Data fitted using T200 analysis software. Figure generated using Prism.
E. Crystal lattice of six CYRI- BΔN:Rac1Q61L molecules. One molecule is shown in solid colour, the remaining five at 50% transparency. Rac1 coloured in green, RBD in blue and the Ratchet subdomain in orange.
F. Linker connecting the C-terminus of CYRI-BΔN to the N-terminus of Rac1Q61L shown in dotted red lines.

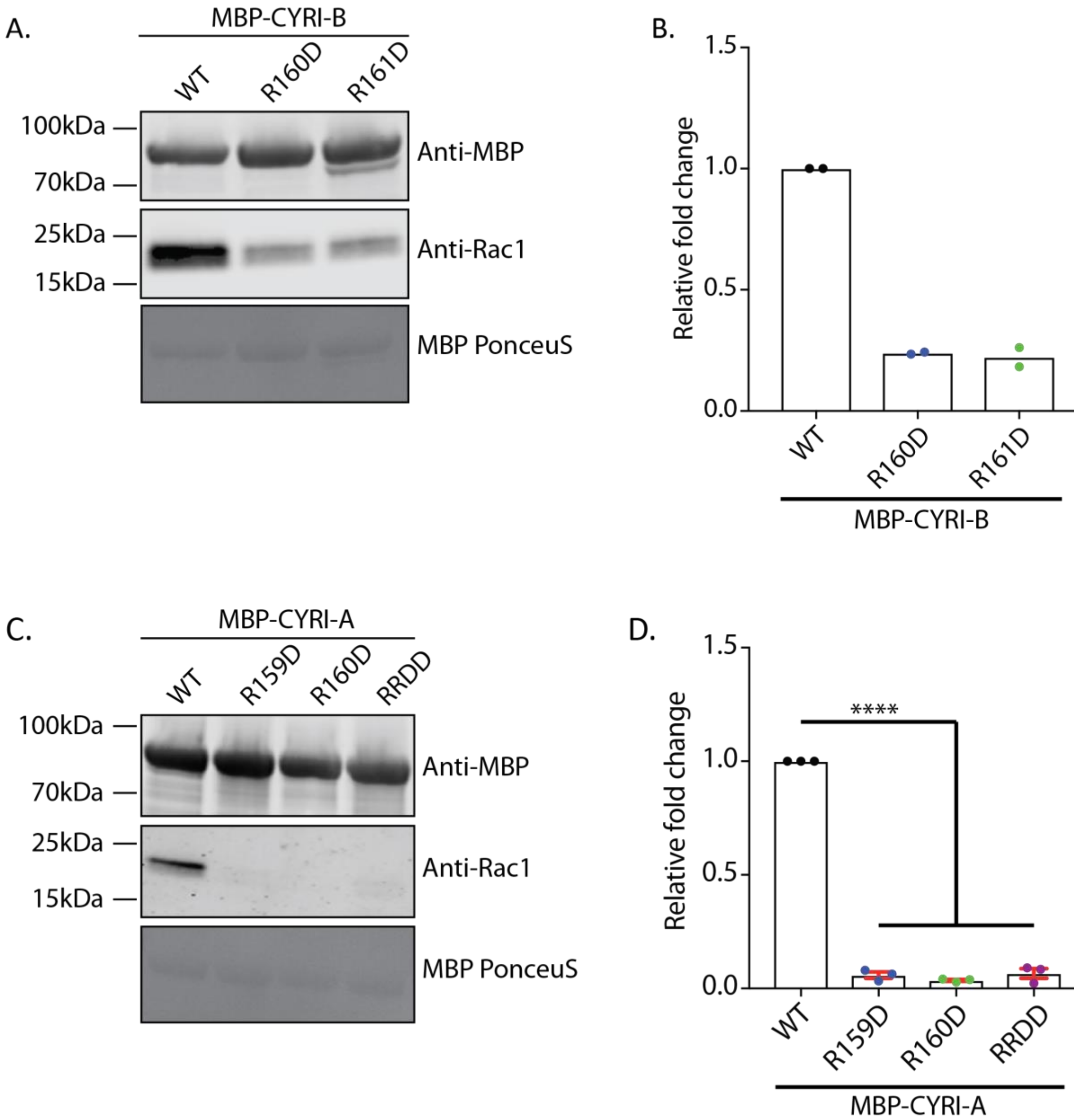

Supplementary Figure 4. Related to Figure 3, 4. The two conserved arginine in CYRI-A and CYRI-B are important for active Rac1 interaction.

A. Pulldown assay using immobilised MBP-CYRI-B shows single arginine mutants could dramatically disrupt their interaction with active Rac1.
B. Quantification shows dramatic decreases in active Rac1 binding to single mutant CYRI-B compared to its WT.
C. Pulldown assay using immobilised MBP-CYRI-A shows both single arginine mutants and double mutant could dramatically disrupt their interaction with active Rac1.
D. Quantification shows dramatic decreases in active Rac1 binding to mutant CYRI-A compared to its WT.

A.

```
CYFIP1      MAAQVTLEDALSNVDLLEELPLPDQQPCIEPPPSSLLYQPNFNTNFEDRNAFVTGIARYI      60
CYFIP2      MTTHVTLEDALSNVDLLEELPLPDQQPCIEPPPSSIMYQANFDTNFEDRNAFVTGIARYI      60
CYRI-A      ------------MGNLLKV------LT-R-----EIENYPHFFLDFENAQPTEGEREIWN      36
CYRI-B      ------------MGNLLKV------LTCT-----DLEQGPNFFLDFENAQPTESEKEIYN      37
                        :**:                    .:    :*  :**: :        :

CYFIP1      EQATVHSSMNEMLEEGQEYAVMLYTWRSCSRAIPQVKCNEQPNRVEIYEKTVEVLEPEVT      120
CYFIP2      EQATVHSSMNEMLEEGHEYAVMLYTWRSCSRAIPQVKCNEQPNRVEIYEKTVEVLEPEVT      120
CYRI-A      -------QISAVLQDSESILADLQAYKGAGPEIR--DAIQNPNDIQLQEKAWNAVCPLVV      87
CYRI-B      -------QVNVVLKDAEGILEDLQSYRGAGHEIR--EAIQHPADEKLQEKAWGAVVPLVG      88
                   .:. :*::..     * :::...  *   .. ::*   :: **:  .: * *

CYFIP1      KLMNFMYFQRNAIERFCGEVRR--------LCHAERRKDFVSEAYLITLGKFINMFAVLD      172
CYFIP2      KLMKFMYFQRKAIERFCSEVKR--------LCHAERRKDFVSEAYLLTLGKFINMFAVLD      172
CYRI-A       RLKRFYEFSIRLEKALQSLLESLTCPPYTPTQHLEREQAL-----AKEFAEILHFTLRFD     142
CYRI-B       KLKKFYEFSQRLEAALRGLLGALTSTPYSPTQHLEREQAL-----AKQFAEILHFTLRFD     143
            :* .*  *. .    : . :             * **.: :         :.:::::   :*

CYFIP1      ELKNMKCSVKNDHSAYKRAAQFLRKMADPQSIQE------SQNLSMFLANHNKITQSLQQ      226
CYFIP2      ELKNMKCSVKNDHSAYKRAAQFLRKMADPQSIQE------SQNLSMFLANHNRITQCLHQ      226
CYRI-A      ELKMRNPAIQNDFSYYRRTISRNRINNMHLDIENEVNNEMANRMSLFYAEATPMLKTLSN      202
CYRI-B      ELKMTNPAIQNDFSYYRRTLSRMRINNVPAEGENEVNNELANRMSLFYAEATPMLKTLSD      203
            ***  : :::**.* *:*: .  *      . ::      ::.:*:* *: . : : * :

CYFIP1      Q-LE--------VISGYEELLADIVNLCVDYYENRMY---LTPSEKHMLLKVMGFGLYLM      274
CYFIP2      Q-LE--------VIPGYEELLADIVNICVDYYENKMY---LTPSEKHMLLKVMGFGLYLM      274
CYRI-A      ATMHFVSENKTLPIENTTDCLSTMTSVCKVMLETPEYRSRFTSEETLMFCMRVMVGVIIL      262
CYRI-B      ATTKFVSENKNLPIENTTDCLSTMASVCRVMLETPEYRSRFTNEETVSFCLRVMVGVIIL      263
              .         * .  : *: :..:*    *.  *   :* .*.  :    : .*: ::

CYFIP1      DGSVSNIYKLDAKKRINLSKIDKYFKQLQVVPLFGDMQIELARYIKTSAHYEENKSRWTC      334
CYFIP2      DGNVSNIYKLDAKKRINLSKIDKFFKQLQVVPLFGDMQIELARYIKTSAHYEENKSKWTC      334
CYRI-A      YDHVHPVGAFCKTSKIDMKGCIKVLKEQAPDSVEG--LLNALRFTT--KHLND-------      311
CYRI-B      YDHVHPVGAFAKTSKIDMKGCIKVLKDQPPNSVEG--LLNALRYTT--KHLND-------      312
             . *  :  :  ..:*::.   * :*:      : *   ::  *: .   * ::

CYFIP1      TSSGSSPQYNICEQMIQIREDHMRFI    360
CYFIP2      TQSSISPQYNICEQMVQIRDDHIRFI    360
CYRI-A      -----ESTSKQIRAMLQ---------    323
CYRI-B      -----ETTSKQIKSMLQ---------    324
                .   :  . *:*
```

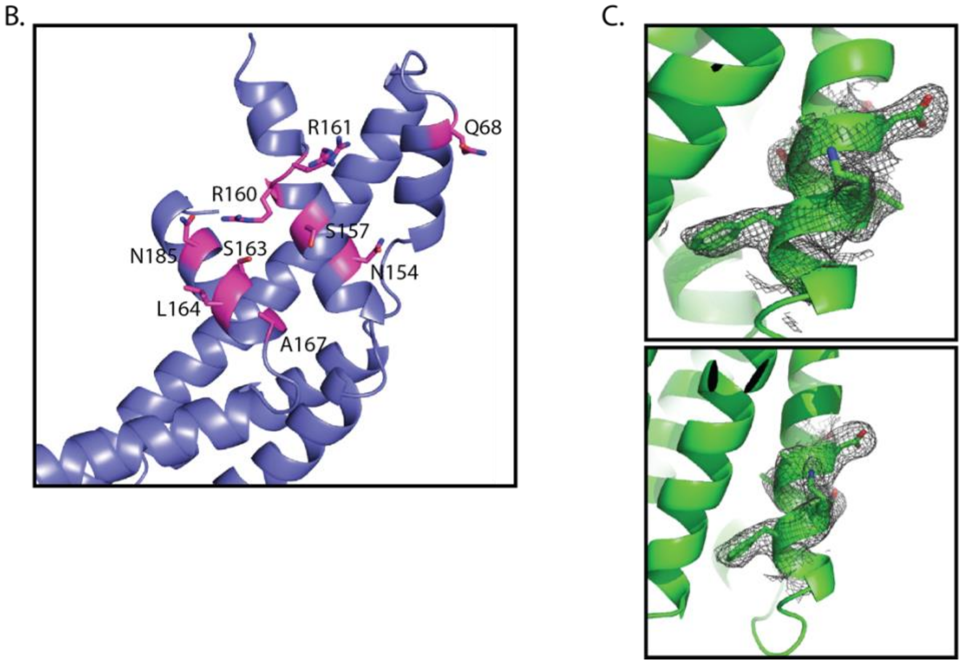

Supplementary Figure 5. Related to Figure 7. Identification of a minimal DUF1394 domain Rac1 binding site.

A. Sequence alignment of human CYFIP1, CYFIP2, CYRI-A and CYRI-B. Residues with conserved biochemical properties at the binding interface are highlighted in red boxes.
B. Conserved residues highlighted on CYRI-B surface in pink and stick form.
C. Representative electron density for both CYRI-BΔN (left) and CYRI-BΔN:Rac1 complex (right). Electron density contoured to 1sigma.

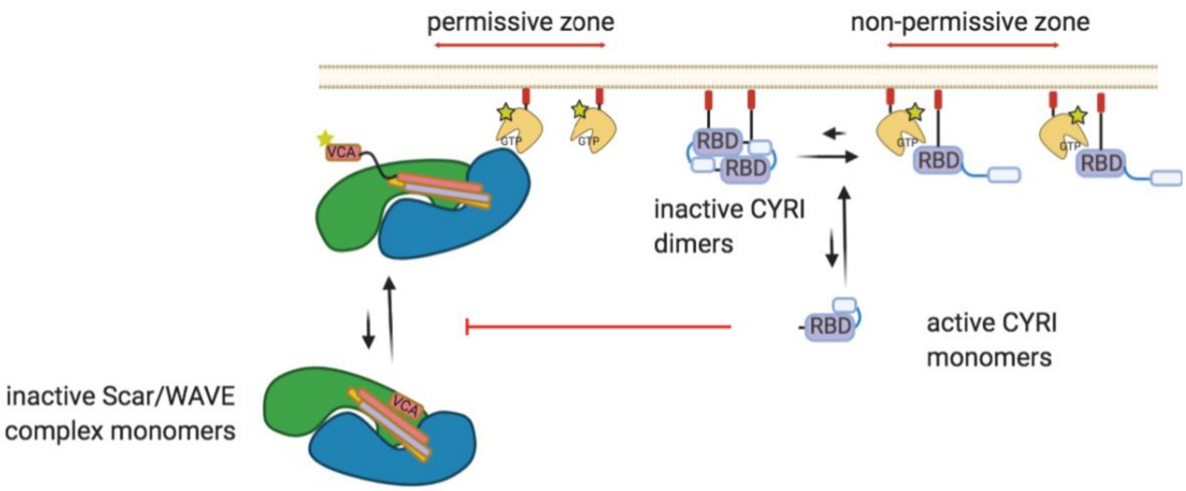

Supplementary Figure 6. Related to Figure 1 to 7. Model for CYRI regulation of active Rac1.

CYRI is recruited by active Rac1 to the plasma membrane. Rac1 binding to the Rac binding subdomain can induce a conformational change displacing the Ratchet subdomain of CYRI. When CYRI binds all active Rac1, a local zone of plasma membrane becomes non-permissive and the Scar/WAVE complex is inhibited. When high local concentrations of CYRI are achieved, an autoinhibited CYRI dimer can be formed, releasing Rac1 to activate the Scar/WAVE complex. Transient interactions between Rac1 and CYRI promote an active feedback loop that can propagate dynamic actin waves, driving protrusions and migration.

	CYRI-BΔN (PDB: 7AJL)	CYRI-BΔN:Rac1 complex (PDB: 7AJK)
Wavelength (Å)	0.97	0.97
Resolution range	41.74 - 2.37 (2.45- 2.37)	55.45 - 3.10 (3.21 - 3.10)
Space group	P 1 21 1	P 62 2 2
Unit cell	44.73 166.68 45.13<br>90.0 112.3 90.0	81.87 81.87 355.87<br>90.0 90.0 120.0
Total reflections	763899	14916
Unique reflections	24723	13492
Multiplicity	23.0 (21.5)	10.6 (11.3)
Completeness (%)	99.94 (100.00)	97.82 (99.77)
Mean I/sigma(I)	25.0 (3.5)	18.7 (3.6)
Wilson B-factor	62.54	88.66
R-merge	7.2 (86.4)	9.2 (66.2)
R-meas	7.5 (90.8)	9.6 (68.1)
CC1/2	0.97 (0.97)	0.99 (0.92)
Reflections used in refinement	24710	13490
Reflections used for R-free	1242	672
R-work	23.9	24.7
R-free	27.2	29.2
No. non-hydrogen atoms	4729	3603
Macromolecules	4568	3558
Solvent	161	12
RMS(bonds)	0.013	0.013
RMS(angles)	1.54	1.54
Ramachandran favoured (%)	95.9	94.7
Ramachandran allowed (%)	4.1	5.1
Ramachandran outliers (%)	0.0	0.2
Rotamer outliers (%)	7.2	6.0
Clashscore	15.6	14.1
Average B-factor	84.5	86.9
Macromolecules	84.9	87.0
Solvent	74.2	65.5

TableS1. Related to Figure 1 and 2. Crystallographic table of statistics

REAGENT or RESOURCE	SOURCE	IDENTIFIER
Oligonucleotides		
CYRI-A-FLAG For:TCAACGGGATCCGCCACCA TGGGAAACTTGCTCAAAGTC	This work/Thermofisher	N/A
CYRI-A-FLAG Rev: TCAACGCTCGAGTTACTTGTCGT CATCGTCTTTGTAGTCCTGAAGCATC GCTCGAATCTG	This work/Thermofisher	N/A
P17-HA-CYRI-B For:TACCCATACGATGTTCCAGAT TACGCTGCTAGCAATTTTTTCCTTG ATTTTGAAAATGCCCAGCC	This work/Thermofisher	N/A
P17-HA-CYRI-B Rev:AGCGTAATCTGGAACATCGTATGGGTACT GAGTGGCCCCTGCTCAAGGTC	This work/Thermofisher	N/A
GST-hRac1Q61L-S41A For: TGACAATTATGCTGCCAATGTTATG	This work/Thermofisher	N/A
GST-hRac1Q61L-S41A Rev: AAGACAGTAGGGATATATTC	This work/Thermofisher	N/A
MBP-mCYRI-B-S157A For: AAATGACTTCGCCTACTACAGAAGAACATTG	This work/Thermofisher	N/A
MBP-mCYRI-B-S157A Rev: TGTATGGCAGGATTTGTC	This work/Thermofisher	N/A
MBP-mCYRI-B-S157R For: AAATGACTTCAGGTACTACAGAAG	This work/Thermofisher	N/A
MBP-mCYRI-B-S157R Rev: TGTATGGCAGGATTTGTC	This work/Thermofisher	N/A
MBP-mCYRI-B-Q153R For: TCCTGCCATACGAAATGACTTCAG	This work/Thermofisher	N/A
MBP-mCYRI-B-Q153R Rev: TTTGTCATCTTGAGCTCATC	This work/Thermofisher	N/A
MBP-HA-mCYRI-B For: CGCGGATCCTATCCGTATGATGT TCCGGATTATGCAATGGGGAATCTTC TTAAAGTTTTGACATGC	This work/IDT	N/A
MBP-HA-mCYRI-B Rev: AAGGAAAAAAGCGGCCGCTTAC TGCAGCATGGACCTAATTTGC	This work/IDT	N/A
Mouse CYRI-BΔN For: CGCGGATCCGCCCAGCCTACA GAGTCTGAGAAG	This work/IDT	N/A
Mouse CYRI-BΔN Rev: CCGGAATTCTTACTATTGCAGCAT GGATTTAATTTGCTTGG	This work/IDT	N/A
MBP-mCYRI-A For: CGTATTGGATCCATGGGAAACTTG CTCAAAGTC	This work/Thermofisher	N/A
MBP-CYRI-A Rev: CGTATTGAAGCTTCTACTGAAGC ATCGCTCGAAT	This work/Thermofisher	N/A
MBP-CYRI-A-R159D For: CAGTTACTATGACAGAACAATAAG TCGTAACCG	This work/Thermofisher	N/A
MBP-CYRI-A-R159D Rev: AAGTCATTCTGAATAGCG	This work/Thermofisher	N/A
MBP-CYRI-A-R160D For: TTACTATCGAGACACAATAAGTCG TAACC	This work/Thermofisher	N/A

MBP-CYRI-A-R160D<br>Rev: CTGAAGTCATTCTGAATAGC	This work/Thermofisher	N/A
MBP-CYRI-A-RRDD<br>For: CAGTTACTATGACGACACAAT AAGTCGTAACCGTATC	This work/Thermofisher	N/A
MBP-CYRI-A-RRDD<br>Rev: AAGTCATTCTGAATAGCG	This work/Thermofisher	N/A

TableS2. Related to Key Resource Table. Oligonucleotides
